# N6-methyladenosine: a key regulator in ocular disease mechanisms and treatment

**DOI:** 10.1038/s41420-025-02867-1

**Published:** 2025-11-28

**Authors:** Yumeng Lin, Liuzhi Zeng, Yunfeng Zhang, Yating Liu, Jun Wang, Chen Xu, Luze Liu, Ping Yu, Zhongyu Han, Sizhen Li, Qingsong Yang

**Affiliations:** 1 Department of Nanjing Tongren Eye Center, Nanjing Tongren Hospital, School of Medicine, Southeast University, Nanjing, China; 2 Department of Ophthalmology, Chengdu First People’s Hospital, Chengdu, China; 3 School of Medicine, Southeast University, Nanjing, China

**Keywords:** RNA splicing, Eye diseases, Epigenetics

## Abstract

N6-methyladenosine (m6A), a pivotal RNA modification, has garnered considerable attention in cell biology and disease research. m6A plays a critical role in the regulation of gene expression, cell proliferation, differentiation, and apoptosis, with particular relevance to the onset and progression of ocular diseases. This review examines the current research on m6A in ocular diseases, including keratitis, cataracts, glaucoma, retinopathy, thyroid ophthalmopathy, and ocular tumors, highlighting its functional significance and potential mechanisms in these conditions. Recent studies suggest that m6A modification influences cellular fate and pathophysiological processes by modulating the expression of key genes. However, a deeper understanding of the precise mechanisms underlying m6A action in ocular diseases is still needed. By synthesizing the existing literature, this review seeks to offer novel insights and identify potential therapeutic targets, thereby advancing clinical applications for ocular disease treatment.

## Facts

1. The molecular mechanisms by which m6A modification regulates ocular diseases remain to be fully elucidated. While current research has revealed that m6A modification influences cellular fate and pathophysiological processes through modulating the expression of key genes, the specific regulatory mechanisms of m6A modification in different types of ocular diseases require further exploration. Clarifying these mechanisms will provide a more solid theoretical foundation for the treatment of ocular diseases.
2. The dynamic regulatory mechanisms of m6A modification in ocular diseases remain unclear. m6A modification is a dynamic and reversible process influenced by various factors such as environmental conditions and disease progression. However, the dynamic changes in m6A modification during the onset and progression of ocular diseases, as well as their regulatory mechanisms, are still poorly understood. Future research should focus on investigating the dynamic regulatory patterns of m6A modification in ocular diseases and the mechanisms of interaction between m6A modification and other signaling pathways.
3. The potential of m6A modification as a therapeutic target for ocular diseases requires validation. Current research has identified some key m6A regulatory proteins and related signaling pathways involved in ocular diseases, but the safety and efficacy of therapies targeting m6A modification need to be further evaluated. In-depth studies on the therapeutic potential of m6A modification will help develop novel therapeutic strategies for ocular diseases and advance their clinical applications.
4. The interplay between m6A modification and other epigenetic modifications in ocular diseases is worth exploring. Epigenetic modifications such as DNA methylation and histone modification play significant roles in the regulation of ocular diseases. The relationship between m6A modification and other epigenetic modifications and their combined effects in ocular diseases remains largely unknown. Future research should investigate the synergistic or antagonistic effects of m6A modification with other epigenetic modifications to uncover their complex regulatory networks in ocular disease pathogenesis.

## Introduction

Epigenetic modifications, including DNA methylation, RNA modification, histone modification, and chromatin remodeling, regulate gene expression through chemical or structural alterations without changing the underlying DNA sequence [1]. Among these, RNA methylation—an essential and reversible form of RNA modification—has garnered considerable attention due to its pivotal role in post-transcriptional regulation [2]. Notably, N6-methyladenosine (m6A) modification, the most prevalent base methylation in mRNA, has attracted significant interest in light of studies on m6A content expression, the distribution of its modifiers in human tissues, and its involvement in cellular functions, as well as its potential as a biomarker [3, 4]. Under physiological conditions, m6A modification regulates gene expression, cell differentiation, and tissue homeostasis, ensuring normal cellular function and development. In pathological contexts, dysregulation of m6A modification is closely associated with the onset and progression of various diseases, affecting processes such as cell proliferation, apoptosis, inflammatory responses, and cell-cell interactions, thereby driving disease progression [5, 6].

**Fig. 1:**
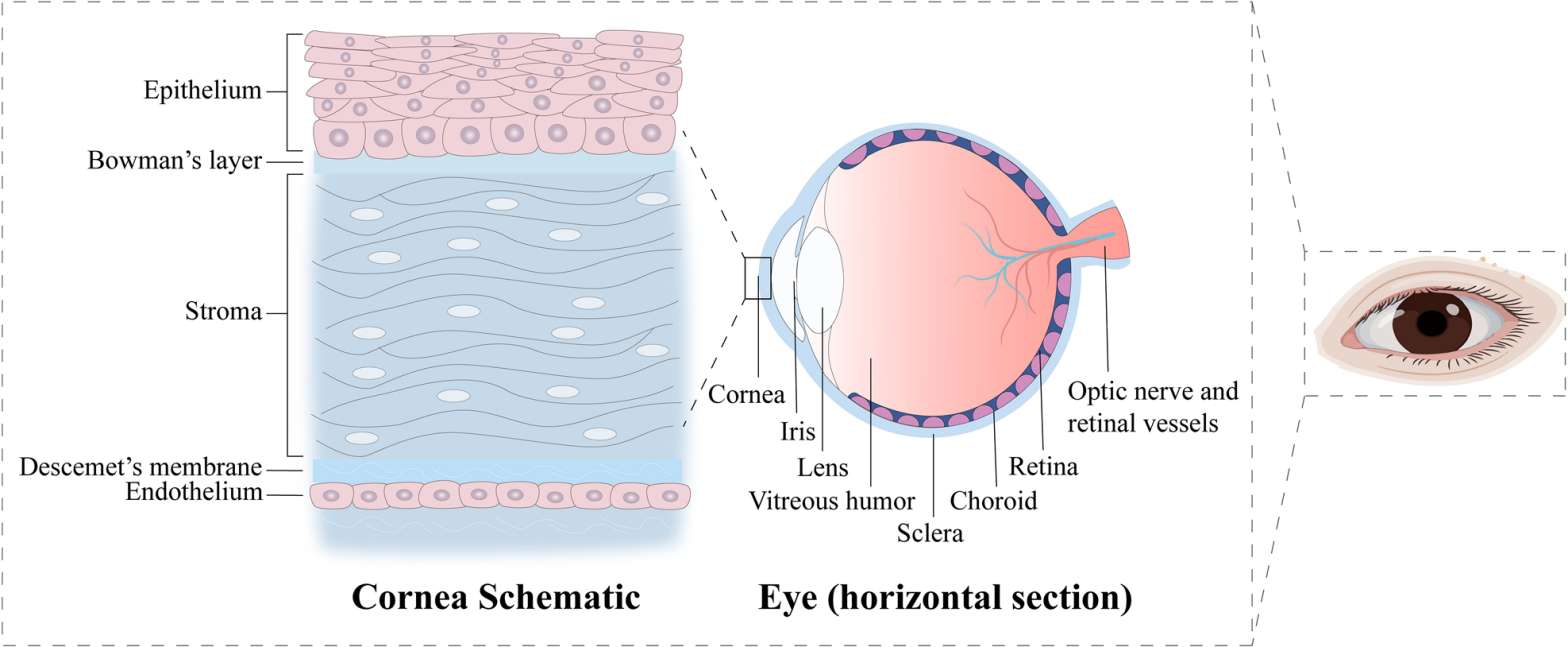
The structure of the eye and the layering of the cornea. As an important part of the visual organ, the eye includes the cornea, lens, vitreous body, retina, optic nerve, etc. The cornea is composed of five different layers: the epithelial cell layer, Bowman’s layer, corneal stromal layer, Descemet’s membrane, and endothelial cell layer. Each layer is essential for the function of the cornea, not only as the first mechanical and immunological barrier of the eye, but also responsible for the transmission and convergence of external light to the retina to produce vision.

The eye is a precise optical organ composed of structures such as the cornea, lens, and retina that are responsible for focusing light and converting it into neural signals to transmit to the brain (Fig. 1). Eye diseases represent a leading cause of visual impairment and blindness, with their pathogenesis intricately linked to epigenetic modifications [7]. Recent studies have highlighted the association between DNA and RNA methylation and the pathological processes of several common ocular diseases [8, 9]. In particular, m6A modification exhibits significant alterations in the cornea, retina, and choroid under pathological conditions, offering new insights into the pathophysiological mechanisms of ophthalmic diseases [10]. Given its importance, investigating RNA methylation in ocular diseases is essential, as it lays a crucial foundation for identifying potential gene therapy targets.

This paper aims to introduce the fundamental concepts, mechanisms, and detection methods of m6A modification. Furthermore, it investigates its regulatory role and potential therapeutic implications in various ocular diseases, including corneal diseases, cataracts, glaucoma, uveitis, retinopathy, Traumatic Optic Neuropathy (TON), thyroid eye disease (TED), myopia, and ocular tumors. Finally, the paper addresses future research directions and the challenges that must be overcome to deepen our understanding of m6A modification in ophthalmic diseases.

## M6A modification

N6-methyladenosine (m6A) modification is one of the most prevalent RNA modifications in eukaryotes. It regulates gene expression by adding a methyl group to the nitrogen atom at the N6 position of adenosine in both mRNA and non-coding RNA. m6A is widespread across various organisms, including viruses, yeast, plants, insects, and mammals, occurring every 700–800 base pairs on average in polyadenylated RNA, and at lower frequencies in other types of RNA [11, 12]. In the human genome, over 12,000 m6A loci have been identified, primarily located in the coding regions and 3’ untranslated regions (3’ UTR) of approximately 7000 genes. The consensus sequence for these loci is RRACH (where R = G/A and H = A/C/U) [13, 14]. M6A modification is a dynamic and reversible process that plays a central role in post-transcriptional regulation, including RNA splicing, stability, trafficking, translation, and degradation. It influences various biological processes, such as cell differentiation, development, immune response, and neurological function [15, 16]. Furthermore, dysregulation of m6A modification is closely associated with the onset and progression of various diseases, including cancer, neurological disorders, metabolic diseases, and immune-related conditions [17, 18]. The dynamic regulation of m6A modification is controlled by three classes of enzymes: m6A methyltransferases (referred to as “writers”), demethylases (“erasers”), and m6A-binding proteins (“readers”), which are responsible for adding, removing, and recognizing the m6A modification, respectively (Table 1).

**Table 1:** The roles of m6A regulators in RNA metabolism.

Type	Proteins	Family	Location	Functions	Ref
Writers	METTL3	Methyltransferase family	Cytoplasm and nucleus	Regulates RNA stability, translation efficiency, and splicing by catalyzing m6A modification	[265]
	METTL14	Methyltransferase family	nucleus	Significantly enhances catalytic activity by stabilizing METTL3 conformation and participating in RNA substrate recognition	[266]
	METTL16	Methyltransferase family	Cytoplasm and nucleus	Specifically adds m6A modifications to various RNA molecules	[267]
	RBM15	RNA-binding protein family	Nucleus	Binds the m6A complex and recruits it to specialized RNA sites	[268]
	WTAP	Wilms tumor-associated protein family	Nucleus	Interacts with METTL3 and METTL14 to regulate m6A modification complex activity	[269]
	VIRMA	Methyltransferase complex	Nucleus	Guides writers to specific RNA regions	[270]
	ZC3H13	CCCH-type zinc finger protein family	Nucleus	Bridges RBM15 to WTAP, ensuring nuclear localization of the m6A methyltransferase complex	[271]
Erasers	FTO (ALKBH9)	Alpha-ketoglutarate-dependent dioxygenase family	Nucleus	Demethylates m6A by oxidizing it to hm6A and then to f6A	[272]
	ALKBH5	Alpha-ketoglutarate-dependent dioxygenase family	Nucleus	Demethylates m6A by directly removing methyl groups from methylated adenosine	[273]
	ALKBH3	Alpha-ketoglutarate-dependent dioxygenase family	Cytoplasm and nucleus	Facilitates DNA repair, tRNA demethylation, and enhances protein translation efficiency	[274]
Readers	YTHDF1	YTH domain protein family	Cytoplasm	Interacts with eIF3 to direct mRNA into the translation initiation complex, promoting translation initiation	[275]
	YTHDF2	YTH domain protein family	Cytoplasm	The C-terminal YTH domain recognizes m6A sites and the N-terminus binds to CNOT1, recruiting the CCR4-NOT complex to transport m6A-modified RNA to P-bodies for accelerated degradation	[276]
	YTHDF3	YTH domain protein family	Cytoplasm	Collaborates with YTHDF1 and YTHDF2 to promote translation efficiency of m6A-modified mRNAs	[277]
	YTHDC1	YTH domain protein family	Nucleus	Interacts with SRSF3, NXF1, and TREX complexes to promote nuclear export and splicing regulation of mRNAs	[278]
	YTHDC2	YTH domain protein family	Cytoplasm	Interacts with 5′3′ exoribonuclease 1 (XRN1) to promote degradation of m6A-modified mRNAs while enhancing translation efficiency	[279]
	IGF2BP1	Insulin-like growth factor 2 mRNA-binding protein family	Cytoplasm	Enhances mRNA stability and translation	[280]
	IGF2BP2	Insulin-like growth factor 2 mRNA-binding protein family	Cytoplasm	Enhances mRNA stability and translation	[281]
	IGF2BP3	Insulin-like growth factor 2 mRNA-binding protein family	Cytoplasm	Enhances mRNA stability and translation	[282]
	HNRNPC	Heterogeneous nuclear ribonucleoprotein family	Nucleus	Regulates mRNA abundance and splicing	[283]
	HNRNPG	Heterogeneous nuclear ribonucleoprotein family	Nucleus	Regulates mRNA abundance and splicing	[284]
	HNRNPA2B1	Heterogeneous nuclear ribonucleoprotein family	Nucleus	Recognizes m6A core motif RGAC and regulates RNA selective cleavage in a METTL3-dependent manner	[285]
	eIF3	Eukaryotic initiation factor family	Cytoplasm	Enhances the assembly of translation initiation complexes, regulating translation efficiency	[286]

*CNOT1* CCR4-NOT transcription complex subunit 1, *f6A* N6-formyladenosine, *hm6A* N6-hydroxymethyladenosine, *NXF1* nuclear RNA export factor 1, *P-body* processing body, SRSF3 serine/arginine-rich splicing factor 3, *TREX* the three-prime repair exonuclease, *XRN1* 5′-3′ Exoribonuclease 1.

### Writers

The m6A “writer” comprises an m6A methyltransferase complex (MTC), consisting of core members such as methyltransferase-like 3 (METTL3), METTL14, and Wilms tumor 1-associated protein (WTAP), along with METTL16, zinc finger CCCH-containing type 13 (ZC3H13), RNA-binding methylprotein 15 (RBM15), and vir-m6A motif transferase-associated protein (VIRMA) [19]. METTL3 serves as the major catalytic subunit, transferring methyl groups from S-adenosylmethionine (SAM) to adenosine on RNA [20]. METTL14 enhances METTL3’s catalytic activity by stabilizing its conformation and facilitating RNA substrate recognition [21]. The METTL3/14 complex predominantly recognizes the GGACU consensus sequence. Although WTAP lacks intrinsic methyltransferase activity, it facilitates m6A deposition by interacting with the METTL3-METTL14 complex and plays a key regulatory role in RNA targeting and splicing [22]. VIRMA guides the METTL3/METTL14/WTAP complex to mediate region-specific mRNA methylation within the 3’ untranslated region (3’ UTR) and near the stop codon [23]. ZC3H13 ensures the nuclear localization of the MTC by bridging RBM15 to WTAP [24, 25].

### Erasers

m6A modification on RNA can be reversed by demethylases, often referred to as “Erasers,” with Fat mass and obesity-associated protein (FTO) and AlkB homolog 5 (ALKBH5) being the two primary RNA demethylases. FTO was the first identified m6A demethylase, achieving demethylation by oxidizing m6A to the intermediate N6-hydroxymethyladenosine (hm6A), which is further oxidized to N6-formyladenosine (f6A) and ultimately decomposes into adenosine (A) in aqueous solution [26, 27]. In contrast, ALKBH5, a nuclear protein that binds single-stranded nucleotides, catalyzes m6A demethylation by directly removing methyl groups from methylated adenosine in m6A, unlike FTO, which operates through an oxidative process [28]. Recently, a novel m6A demethylase, ALKBH3, has been identified and shown to be involved in tRNA demethylation [29]. FTO, ALKBH5, and ALKBH3 all belong to the alpha-ketoglutarate-dependent dioxygenase family, and their demethylation process utilizes 2-oxoglutarate (2OG) and Fe (II) as cofactors [29, 30].

### Readers

The “reader” of m6A mainly consists of the YT521B homology (YTH) domain family (YTHDF1/2/3, YTHDC1/2), insulin-like growth factor 2 mRNA-binding proteins (IGF2BP1/2/3), heterokaryotic nuclear factor RNA proteins (HNRNPC, HNRNPG, and HNRNPA2B1), and eukaryotic initiation factor 3 (eIF3).

The YTH domain is a conserved m6A-binding domain that selectively interacts with the m6A site in RNA. These molecules represent the most crucial “readers” of m6A modification [31]. As the first identified reader, the C-terminal YTH domain of YTHDF2 recognizes a specific m6A locus, while its N-terminal domain binds to the SH domain of the CCR4-NOT transcription complex subunit 1 (CNOT1), facilitating the recruitment of the CCR4-NOT deadenylase complex. This interaction directs m6A-modified RNA to the processing body (P-body), promoting the deadenylation and degradation of its poly(A) tail, thereby enhancing RNA degradation [32, 33]. YTHDF1, specifically recognizing m6A-modified mRNAs through its YTH domain, interacts with the translation initiation factor eIF3 to direct these mRNAs into the translation initiation complex, thereby promoting translation initiation [34]. Additionally, the regulation of YTHDF1 depends on an eIF4G (eukaryotic initiation factor 4 G)-mediated loop structure, which enhances the efficiency of translation initiation for m6A-modified mRNAs, significantly boosting translation efficiency [35]. In contrast, YTHDF3 collaborates with the other two paralogues to regulate the translation and degradation of mRNA containing m6A in the cytoplasm [36].

YTHDC1 collaborates with nuclear RNA export factor 1 (NXF1) and the three-prime repair exonuclease (TREX) mRNA export complex, interacting with serine/arginine-rich splicing factor 3 (SRSF3) to facilitate mRNA nuclear export and regulate splicing [37, 38]. As a 3’/5’ RNA helicase, YTHDC2 promotes the degradation of m6A-modified mRNAs through its helicase activity. It also enhances mRNA stability by interacting with 5′–3′ exoribonuclease 1 (XRN1), thereby improving the translational efficiency of target mRNAs while simultaneously reducing their overall abundance [39, 40].

**Fig. 2:**
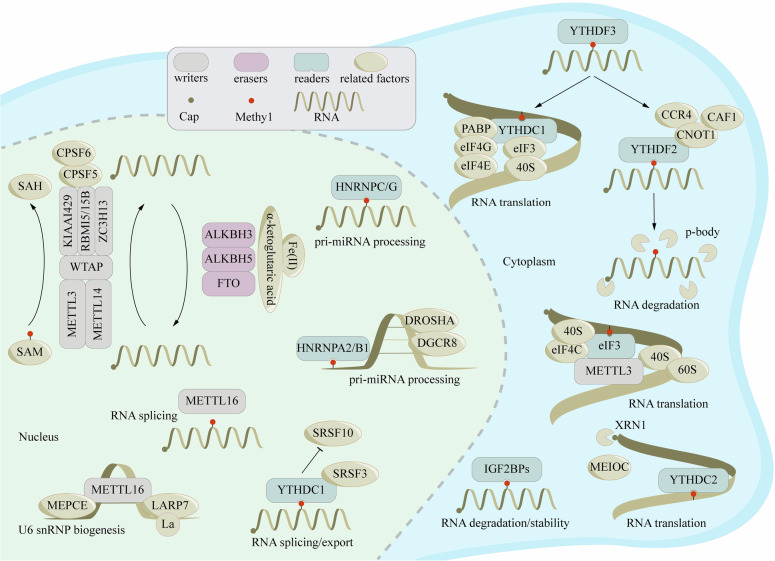
The detailed molecular mechanism of m6A enzymes. The molecular mechanisms of m6A modification involve dynamic interplay among three key groups of enzymes: the “writers,” “erasers,” and “readers.” These enzymes and associated factors work in concert to add, remove, and recognize m6A marks on RNA, thereby regulating various aspects of RNA metabolism. Specifically, m6A modifications influence critical RNA processes such as splicing, nuclear export, translation efficiency, and degradation, ultimately impacting gene expression and cellular function.

In RNA biology, m6A modification profoundly influences the secondary structure of RNA. HNRNPC and HNRNPG regulate mRNA abundance and its splicing process upon recognizing m6A, a mechanism referred to as the “m6A switch.” Additionally, HNRNPC and HNRNPG participate in the processing of precursor mRNAs, thereby affecting their stability, splicing, export, and translation [41, 42]. Moreover, HNRNPA2/B1 recognizes the m6A core motif RGAC and regulates selective RNA cleavage in a METTL3-dependent manner, thus promoting precursor miRNA processing. IGF2BPs recognize m6A-modified mRNAs and enhance mRNA stability and translation by recruiting RNA stabilizers in an m6A-dependent fashion (Fig. 2) [43, 44].

## Techniques for identifying RNA m6A modifications

Studies on RNA modifications date back to the 1970s; however, it was not until 2012 that the combination of RNA immunoprecipitation with next-generation sequencing led to significant advances in m6A research [45]. Initially, identifying the m6A locus posed challenges due to technical limitations. The introduction of second-generation sequencing (seq) marked a milestone in m6A research, enabling substantial progress in the field [13].

MeRIP-seq (also known as m6A-seq) was the first widely used technique for transcriptome-wide mapping of m6A modifications. It employs anti-m6A antibodies to immunoprecipitate methylated mRNA fragments, which are then identified through high-throughput sequencing as enriched genomic regions known as “m6A peaks” [46]. The main advantages of this method include its relatively low RNA input requirement and a well-established experimental workflow, making it suitable for obtaining a broad, discovery-oriented overview of m6A modifications across the transcriptome. However, MeRIP-seq has several notable limitations. Its resolution is low—approximately 100–200 nt—which prevents the precise identification of methylated adenosines and yields only approximate modification regions [47]. More critically, the technique is highly dependent on the quality and specificity of the anti-m6A antibodies [48]. Issues such as non-specific binding to unrelated RNA structures or epitopes, as well as cross-reactivity, can lead to false-positive signals, while insufficient affinity for m6A in certain sequence contexts may result in false negatives. Furthermore, as an enrichment-based rather than an absolute quantitative approach, MeRIP-seq offers limited accuracy in comparing m6A dynamics across different samples.

To address the resolution limitations of MeRIP-seq, PA-m6A-seq was developed. This technique builds upon the MeRIP-seq protocol by incorporating a 4-thiouridine (4SU)-enhanced crosslinking step. A key advantage of PA-m6A-seq is its improved resolution—around 30 nt near the m6A site—enabling more precise mapping of modified regions [49]. The incorporation of 4SU introduces characteristic mutations at crosslinking sites during reverse transcription, providing a clearer molecular footprint of m6A locations. However, a major limitation of this method is its “blind-spot” nature: it can only detect m6A modifications situated near 4SU incorporation sites, thereby restricting its coverage and potentially missing functionally important m6A sites outside these regions. Moreover, like MeRIP-seq, PA-m6A-seq relies on anti-m6A antibodies for immunoprecipitation, meaning it remains susceptible to antibody-related artifacts such as non-specific binding and background bias [50].

The development of m6A individual-nucleotide-resolution crosslinking and immunoprecipitation (miCLIP) marked a major advance by enabling single-base resolution mapping of m6A sites [51]. In miCLIP, UV crosslinking covalently attaches antibodies to m6A-modified residues. During subsequent reverse transcription, the crosslinked antibody residues induce nucleotide misincorporation or truncation events in the cDNA, which are then detected by sequencing to pinpoint m6A sites with high precision. Despite this breakthrough, miCLIP still fundamentally depends on antibody recognition. Incomplete crosslinking efficiency can limit sensitivity, causing some authentic m6A sites to be missed. Additionally, the crosslinking process itself may introduce sequence-specific biases and lead to false-positive identifications. Consequently, even at single-base resolution, potential inaccuracies stemming from antibody specificity remain a concern, underscoring the need for careful data interpretation and validation using orthogonal experimental approaches.

M6A-REF-seq can quantitatively detect m6A loci at single-base resolution and accurately identify them across the entire transcriptome using the RNA endonuclease MazF and antibody-dependent methods [52]. In contrast, DART-seq eliminates the need for antibodies. It employs cytidine deaminase (APOBEC1) and YTH domain fusions to deaminate cytosines adjacent to uracil in m6A, detecting C-to-U editing events via RNA-seq and enabling high-resolution detection of m6A loci [53]. M6A-label-seq introduces N6-allyladenosine (a6A) at the m6A modification site using metabolic markers, which cyclizes to N1, N6-cyclic adenosine (cycA) through iodination. This method induces A-to-C/T/G mutations during reverse transcription, achieving single-base resolution detection at the m6A site [54]. The m6A-SEAL technology specifically labels the m6A site through FTO oxidation and a Dithiothreitol (DTT) chemical reaction, followed by biotin binding for enrichment. This approach enables highly sensitive detection of m6A modification sites across the transcriptome using high-throughput sequencing [55].

Building on m6A-seq, m6A-seq2 introduces multiplex sample mixing and barcode labeling technologies, which enhance experimental throughput and statistical power, improve the quantitative analysis of m6A modification, and enable efficient and accurate detection across multiple samples from a single sample [56]. MiCLIP2 significantly increases the accuracy and sensitivity of m6A detection by optimizing the experimental process and incorporating the machine learning model m6Aboost, while reducing the input material requirements [57]. M6A-LAIC-seq achieves single-nucleotide resolution detection of m6A-modified sites by photocrosslinking to immobilize m6A-modified RNA-protein complexes, followed by enrichment and sequencing using specific antibodies [50]. Techniques such as SELECT, MeRIP-qPCR, and MazF-qPCR are highly sensitive methods for detecting m6A, particularly suited for validation and quantitative analysis of m6A modifications at the single-gene level [52, 58]. SCARLET precisely localizes m6A modification sites through specific cleavage, radiolabeling, ligation-assisted extraction, and Thin-Layer Chromatography (TLC) analysis, without requiring antibodies, making it versatile for various RNA types [59]. In addition, Mass Spectrometry (MS), Dot-blot, and Colorimetry are commonly employed to measure global m6A levels [60, 61] (Table 2).

**Table 2:** Experimental techniques for detecting m6A modifications.

Methods	Principle	Advantages	Disadvantages	Ref
MeRIP-seq/m6A-seq	Antibody-based enrichment of methylated RNA fragments for sequencing.	High-throughput, standardized, genome-wide mapping.	Low resolution, antibody-dependent, high sample demand.	[47]
PA-m6A-seq	4SU and UV crosslinking enhance detection specificity.	Single-base resolution, high accuracy, transcriptome-wide coverage.	Requires 4SU, relative quantification, may miss distant sites.	[49]
m6A-CLIP/miCLIP	UV crosslinking and protease digestion for precise site mapping.	Single-base resolution, high precision, broad coverage.	Complex operation, difficulty in adjacent adenosine positioning.	[51]
m6A-REF-seq	Identifies m6A via MazF enzyme’s cleavage of unmethylated ACA sequences.	Antibody-free, single-base resolution, high sensitivity.	Strict conditions, high sample quality required, motif-dependent.	[52]
DART-seq	APOBEC1-YTH fusion induces C-to-U deamination at m6A sites.	Antibody-free, low RNA input, dynamic monitoring.	Motif-dependent, potential off-target effects, complex engineering.	[287]
m6A-label-seq	Converts m6A sites using methyltransferases for detection.	Single-base resolution, antibody-free, high sensitivity.	Complex processing, labeling efficiency concerns.	[54]
m6A-SEAL-seq	Oxidizes m6A to hm6A, then labels and sequences.	Antibody-free, comprehensive site capture, no sequence bias.	Time-consuming, RNA stability-dependent, chemical interference risks.	[55]
m6A-seq2	Optimized MeRIP-seq with barcoding for quantification.	High-throughput, cost-effective, reproducible.	Antibody-dependent, no resolution improvement.	[56]
miCLIP2	UV crosslinking with machine learning for enhanced detection.	High sensitivity, small input, reduced false positives.	Complex operation, antibody-dependent, computational demands.	[57]
m6A-LAIC-seq	Antibody-based quantification with internal standards.	Transcriptome-wide coverage, quantitative, comparative suitability.	Antibody-dependent, limited resolution, and time-consuming.	[50]
SELECT	Chemical labeling via methyltransferases and RT mutagenesis.	Single-base resolution, high sensitivity, low sample demand.	Complex operation, high cost, enzyme-dependent.	[58]
MeRIP-qPCR	Antibody-based enrichment followed by qPCR.	High specificity, suitable for low-abundance RNA.	Antibody-dependent, background noise susceptibility.	[288]
MazF-qPCR	Detects m6A via MazF cleavage of unmethylated ACA.	Single-base resolution, dynamic analysis, and cost-effectiveness.	Limited to m6ACA sites, primer design required.	[289]
SCARLET	Site-specific cleavage, radiolabeling, and TLC analysis.	Single-base resolution, quantification of modification levels.	Radioactive reagents, time-consuming, and no global analysis.	[59]
MS	Direct analysis of m6A modifications via mass spectrometry.	High sensitivity, suitable for complex samples.	Cannot locate sites precisely, complex preparation, and technical demands.	[290]
Dot-blot	Detects m6A using antibodies or probes on solid-phase carriers.	Simple, low-cost, semi-quantitative.	Cannot locate sites, low sensitivity, and antibody cross-reactivity.	[291]
Colorimetry	Detects total m6A content using specific reagents.	Simple, low-cost, high-throughput.	Cannot locate sites, low sensitivity, and impurity susceptibility.	[292]
TLC	Separates and detects m6A via thin-layer chromatography.	Low-cost, flexible sample processing.	Cannot locate sites, low sensitivity, radiolabeling-dependent.	[27]
m6A-SAC-seq	Enzymatic labeling, chemical transformation, and RT mutagenesis.	Single-base resolution, low sample demand.	Sequence preference, enzyme activity-dependent.	[293]
HPLC-MS	Combines liquid chromatography with mass spectrometry for detection.	Wide analysis range, high separation ability.	Low resolution, antibody-dependent, high sample demand.	[294]
NDRNA-Seq	Nanopore sequencing for direct m6A detection in RNA molecules.	Single-base resolution, direct RNA detection, low sample demand.	Requires 4SU, relative quantification, may miss distant sites.	[295]

*4SU* 4-thiouridine, *MS* mass spectrometry, *TLC* thin-layer chromatography.

## M6A modification in the pathology of ocular disease

The study of m6A modification in ocular diseases is rapidly expanding, yet its precise role in specific ocular conditions remains poorly understood. M6A modification holds significant potential for ophthalmological research, particularly regarding its fundamental pathophysiological functions, such as oxidative stress, angiogenesis, inflammatory responses, and neurodegeneration. These processes are pivotal in the development of ocular diseases, and the regulatory mechanisms of m6A modification in these pathways require further exploration.

### Oxidative stress

Oxidative stress (OS) refers to an imbalance between the oxidative and antioxidant systems within the body, typically characterized by an increased production of Reactive Oxygen Species (ROS) and Reactive Nitrogen Species (RNS). When this imbalance surpasses the body’s antioxidant capacity, it disrupts the intracellular redox balance and results in cellular damage [62].

OS can damage retinal vascular endothelial cells, contributing to retinal vascular diseases such as Age-Related Macular Degeneration (AMD) and Diabetic Retinopathy (DR) [63]. Crystallins, which are major proteins in the lens, play a crucial role in maintaining transparency. Excessive ROS induces conformational changes in crystallin proteins, ultimately leading to cataracts [64, 65]. OS markers, including 8-hydroxy-2′-deoxyguanosine (8OHdG) and malondialdehyde (MDA), are elevated in the serum, aqueous humor, and trabecular meshwork of glaucoma patients. These markers are strongly correlated with elevated intraocular pressure (IOP), visual field defects, and an increased cup-disc ratio (CDR) [66, 67]. Moreover, antioxidants have shown promise in treating dry eye disease. For example, vitamin B12 eyedrops and hyaluronic acid-containing eyedrops have been shown to reduce oxidative stress and improve dry eye symptoms [68]. Recent studies indicate that m6A modification plays a pivotal role in the development of various oxidative stress-related ocular diseases, offering a promising new avenue for treatment based on m6A modulation [69].

### Angiogenesis

Angiogenesis is crucial for wound healing and tissue repair under normal physiological conditions. However, abnormal angiogenesis can lead to severe vision loss and, in some cases, irreversible blindness in ocular diseases [70, 71]. For instance, wet AMD is triggered by excessive activation of vascular endothelial growth factor (VEGF), resulting in abnormal vascular growth, leakage, and macular damage, which contribute to significant visual impairment. While current anti-VEGF therapies slow disease progression, they do not fully reverse visual loss [72, 73]. Similarly, Diabetic Retinopathy (DR) is closely associated with abnormal angiogenesis and can lead to retinal vascular injury due to hyperglycemia (HG), inducing abnormal vessel formation, rupture, and bleeding. These changes result in blinding complications such as retinal edema, vitreous hemorrhage, and retinal detachment [74]. Retinal vein occlusion (RVO) causes retinal hypoxia due to obstructed venous blood flow, stimulating the release of pro-angiogenic factors like VEGF and precipitating abnormal vascular growth. These newly formed blood vessels, characterized by their immaturity, susceptibility to bleeding, and leakage, can further damage retinal structure and impair visual acuity [75, 76].

Hypoxia is a primary driver for the release of pro-angiogenic factors, and recent studies have demonstrated that m6A modification regulates hypoxia-induced angiogenic processes [77]. For example, hypoxia-induced upregulation of METTL3 was observed in endothelial progenitor cells (EPCs), activating the PI3K/AKT signaling pathway, which subsequently promoted EPC-mediated angiogenesis [78]. In contrast, under hypoxic conditions, ALKBH5 expression increases in cardiac microvascular endothelial cells, leading to a reduction in m6A levels. This decrease in m6A triggers WNT5A mRNA degradation, ultimately inhibiting angiogenesis [79]. These findings highlight that m6A-related regulators exert complex effects on angiogenesis, depending on the cellular context.

Previous studies have highlighted the critical role of METTL3-dependent m6A modification in hypoxia-induced pathological ocular angiogenesis, demonstrated through an in vivo ocular neovascularization model [80]. Under hypoxic conditions, METTL3 activity increases, upregulating the protein expression of MMP2 and TIE2 via m6A modification. This, in turn, promotes the migration and tubular formation ability of Retinal Endothelial Cells (RECs), ultimately driving abnormal angiogenesis [81]. Additionally, Cytochrome P450 epoxygenase 2J2 (CYP2J2) overexpression enhances Annexin A1 (ANXA1) protein expression through METTL3-mediated ANXA1 m6A modification, protecting retinal vascular endothelial cells from oxidative stress and preserving the integrity of the Blood-Retinal Barrier (BRB) [82]. Furthermore, FTO regulates endothelial cell (EC) function and Focal Adhesion Kinase (FAK) expression via its demethylase activity and the m6A-YTHDF2-dependent mechanism, contributing to pathological angiogenesis [83].

Angiogenesis is intricately linked to oxidative stress, often resulting from tissue hypoxia and unmet oxygen demands. Under physiological conditions, moderate oxidative stress promotes angiogenesis, playing a vital role in tissue repair and regeneration. However, excessive oxidative stress can lead to abnormal angiogenesis and vascular dysfunction under pathological conditions [84, 85].

### Inflammatory response

Inflammation is the body’s immune response to injury or infection, characterized by redness, swelling, heat, pain, and dysfunction, with the primary aim of clearing pathogens and repairing tissues. In ocular diseases, inflammation activates a variety of cells and signaling pathways that drive pathological processes, with m6A modification playing a central role in regulating these processes.

Feng et al. found that FTO downregulation under diabetic conditions (DM) promotes macrophage polarization toward the M1 type, enhances the expression of inflammatory mediators, and exacerbates diabetic retinopathy (DR)-related microvascular inflammation. Mechanistically, FTO regulates FGF2 mRNA through YTHDF2-dependent m6A modification and activates the PI3K/Akt signaling pathway [86]. Dry eye disease (DED) is often secondary to DM. Guo et al. observed that the expression of WTAP and NEAT1 was upregulated in a mouse model of diabetic dry eye (DMDED), leading to severe corneal injury and inflammation. Knockdown of WTAP/NEAT1 alleviated these symptoms [87]. Moreover, the DM-induced hyperglycemic (HG) environment leads to FTO upregulation, which removes the m6A modification from TNIP1 mRNA, decreasing its stability and reducing TNIP1 protein expression. The reduction of TNIP1 relieves the inhibition of the NFκB signaling pathway, resulting in elevated levels of inflammatory factors (e.g., IL-1β and IL-18) and exacerbating retinal vascular inflammation and endothelial dysfunction [88]. Decreased FTO may promote microglial inflammation in autoimmune uveitis (EAU), suggesting that restoring or activating FTO function could serve as a potential therapeutic strategy for uveitis [89].

### The complexity of m6A regulation

The expanding body of research on m6A in ocular diseases reveals a complex and often context-dependent regulatory landscape. A critical examination of apparent inconsistencies in the functions of individual m6A regulators is essential for a nuanced understanding of their roles in pathophysiology.

The expression and pathological contribution of the demethylase FTO demonstrate striking divergence across different disease environments, sometimes presenting a seeming paradox. In diabetic retinopathy, recent studies have demonstrated that FTO expression is upregulated in endothelial cells under diabetic conditions. This increase, driven by lactate-mediated histone lactylation, promotes angiogenesis, disrupts vascular integrity, and exacerbates inflammation by enhancing the m6A-dependent mRNA stability of targets like CDK2, thereby positioning FTO as a key driver of pathology [90]. In contrast to a hypothetical pro-inflammatory role, accumulating evidence indicates that FTO is downregulated in microglia during uveitis. This downregulation exacerbates the disease by disrupting m6A homeostasis and activating the GPC4/TLR4/NF-κB pathway, positioning FTO as a potential suppressor of microglia-driven inflammation [89].

The role of the methyltransferase METTL3 in inflammatory eye diseases demonstrates a complex, context-dependent pattern. In experimental autoimmune uveitis (EAU), METTL3 expression is significantly decreased in T cells, and its overexpression ameliorates disease by suppressing pathogenic Th17 responses through the ASH1L-YTHDC2 axis, revealing a clear anti-inflammatory function in this specific cellular context [91]. This protective role stands in contrast to its reported functions in broader immune dysregulation, where METTL3 is often associated with suppressing antiviral immunity and disrupting immune tolerance, with its overexpression linked to increased susceptibility to viral infections and autoimmune conditions [92]. These divergent findings underscore that the function of METTL3 is not intrinsic but is determined by the specific disease environment and, critically, the cell type in which it is expressed—demonstrating distinct consequences in lymphoid cells (e.g., T cells) versus myeloid cells.

In conclusion, the seemingly paradoxical functions of m6A regulators like FTO and METTL3 are not experimental artifacts but rather reflections of a sophisticated and dynamic regulatory network. Their ultimate biological output is determined by a confluence of factors, including the specific pathological context, cell type, disease stage, and the particular repertoire of target mRNAs they act upon. Acknowledging and systematically investigating this complexity is a crucial step toward moving from phenomenological observations to a mechanistic understanding that could inform future therapeutic strategies.

## The multifaceted roles of m6A modification in specific ocular disease

**Fig. 3:**
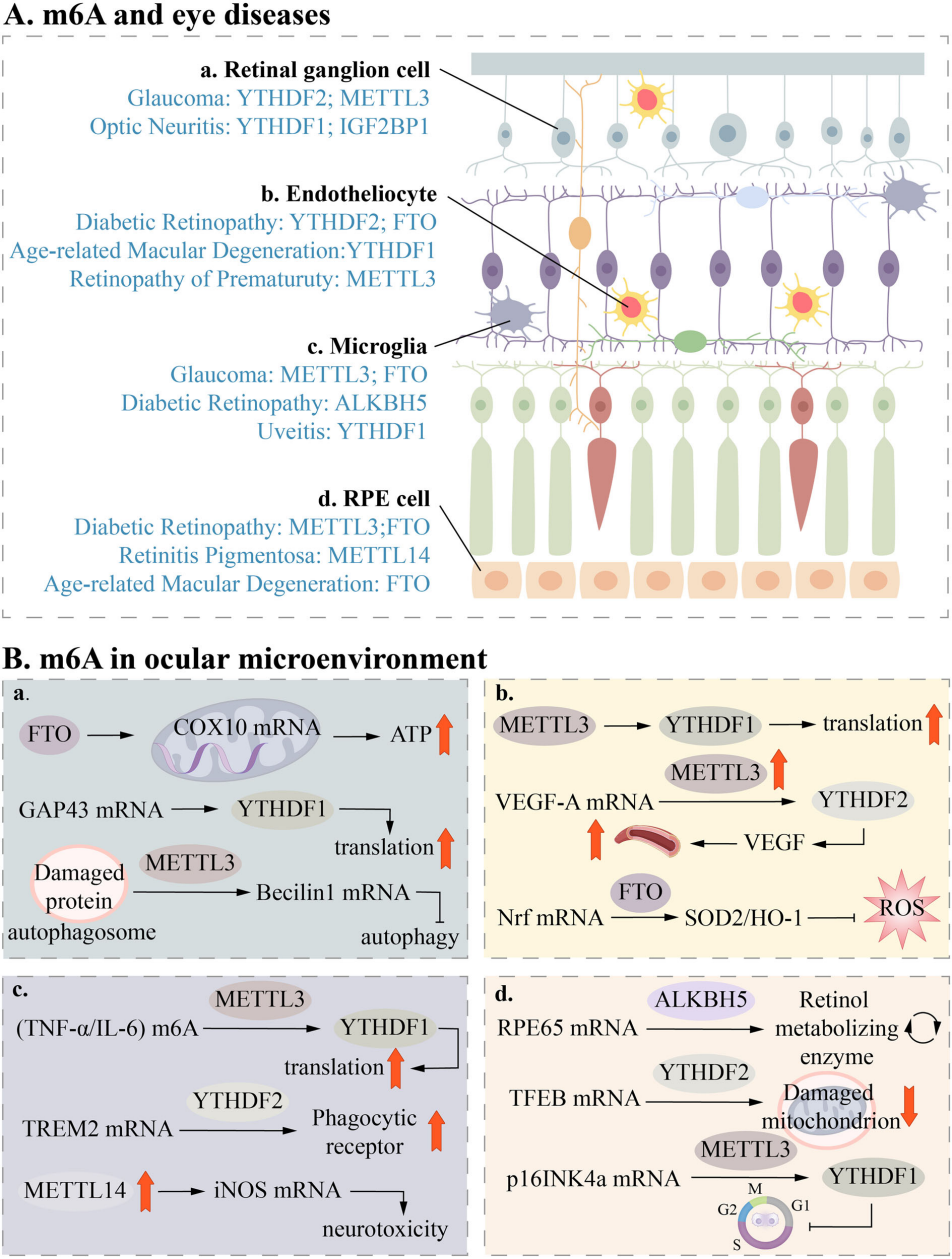
M6A regulatory proteins play a role in the onset and progression of eye diseases. **A** Key regulatory factors of m6A modification in different ocular cell types and related diseases. This includes the expression and function of m6A methyltransferases (METTL3, METTL14), demethylases (FTO, ALKBH5), and reader proteins (YTHDF1, YTHDF2, IGF2BP1) in retinal ganglion cells (glaucoma, optic neuritis), endotheliocyte (diabetic retinopathy, age-related macular degeneration, retinopathy of prematurity), microglia (glaucoma, diabetic retinopathy, uveitis), and RPE cells (diabetic retinopathy, retinitis pigmentosa, age-related maculardegeneration). **B** Molecular regulatory pathways of m6A modification in the ocular microenvironment. a FTO regulates COX10mRNA, affecting ATP production; METTL3 participates in the autophagy process by regulating Beclin1 mRNA. b METTL3/YTHDF1/YTHDF2 and FTO respectively regulate VEGF-A and Nrf pathways, influencing angiogenesis and oxidative stress. c METTL3 mediates inflammatory factors TNF-α/IL-6, and METTL14 is associated with iNOS mRNA expression, participating inneurotoxic responses. d ALKBH5 and YTHDF2 regulate RPE65 and TFEB mRNA, affecting retinol metabolism and mitochondrialfunction; METTL3/YTHDF1 regulate p16INK4a expression, participating in the cell cycle process.

As the core organ of visual perception, the integrity of both the structure and function of the human eye is essential for maintaining clear vision. The complexity of the eye is reflected not only in its intricate anatomy but also in its unique physiological functions and high sensitivity to various internal and external stimuli. In recent years, with the deepening of ocular disease research, m6A modification—a key epigenetic regulatory mechanism—has emerged as an important area of study. M6A modification plays a pivotal role in the pathogenesis of various ocular diseases by regulating gene expression, RNA stability, and translational efficiency. From oxidative stress to neurodegeneration, and from inflammatory responses to angiogenesis, m6A modification exerts diverse regulatory roles in different pathological processes. However, its specific mechanism of action in various ocular diseases remains incompletely understood, and in some cases, it may exert opposing effects. Therefore, a comprehensive investigation into the role of m6A modification in specific ocular diseases will not only illuminate its function in the development of eye diseases but also offer new insights for the development of targeted therapeutic strategies (Fig. 3).

### Anterior segment and adnexal diseases

#### Keratitis

Keratitis is a common corneal disorder, typically characterized by pain, redness, lacrimation, photophobia, and decreased vision, often caused by bacteria, fungi, or viruses [93, 94]. If left untreated, infectious keratitis can result in corneal scarring, leading to opacity and significantly impairing vision [95, 96].

Fungal keratitis (FK) is a severe corneal infectious disease associated with vision loss. It is highly destructive and can result in permanent blindness or even the loss of the eyeball [97]. The main pathogens of FK include Fusarium, Aspergillus, and Candida, with Fusarium solani (F. solani) being the most commonly reported species [98]. In investigating the role of METTL3 in F. solani-induced FK, studies by Hu et al. and Huang et al. have each contributed to this area. Hu et al. focused on global changes in m6A modification and transcriptome analysis, revealing a broad upregulation of m6A modification in corneal tissue following infection. Huang et al. further explored the specific mechanism of METTL3 in the PI3K/AKT signaling pathway and found that METTL3 significantly influences the onset and progression of keratitis by regulating this pathway. The findings of these two studies complement one another, offering a more comprehensive understanding of the role of m6A modification in fungal keratitis. [99, 100]. Moreover, Tang et al. demonstrated that inhibition of METTL3 mitigated the reduction in NFκB signaling, thereby alleviating Fusarium solani keratitis [101]. Therefore, METTL3 may regulate multiple signaling pathways that contribute to the development of FK, making it a potential target for therapeutic strategies.

#### Corneal neovascularization

Corneal neovascularization is typically triggered by factors such as trauma, infection, ischemia, hypoxia, inflammation, or following keratoplasty. It compromises vision by activating pro-angiogenic factors that promote the growth of blood vessels from the limbus to the center of the cornea, leading to the loss of corneal transparency [102].

Yao et al. found that hypoxia significantly increased m6A modification levels in both in vitro cell experiments and in vivo animal models. This increase may influence angiogenesis by regulating the expression of genes involved in the process. Moreover, hypoxia activated Hypoxia-Inducible Factor (HIF), which induced METTL3 expression and promoted angiogenesis. Yao et al. further validated the role of METTL3 in pathological angiogenesis using an alkali burn-induced mouse corneal neovascularization model. METTL3 regulates the expression of Wnt signaling pathway-related genes (e.g., LRP6 and DVL1) through m6A modification, which subsequently affects angiogenesis. In the alkali burn model, specific knockdown of METTL3 reduced the expression of these genes, inhibiting neovascularization [80].

In addition, Dai et al. found that METTL3 knockout mice exhibited a significantly faster rate of corneal injury repair in an alkali burn-induced model, with sodium fluorescein staining showing a markedly increased rate of repair. This result may be linked to METTL3’s regulation of target genes AHNAK and DDIT4 expression through m6A modification, which in turn inhibits the function of limbal stem cells (LSCs). Previous studies have shown that LSCs suppress the formation of vascular endothelial cells under normal conditions, and their loss of function leads to the generation of new blood vessels [103, 104]. Therefore, knockdown of METTL3 may accelerate corneal injury repair and reduce neovascularization by relieving the inhibition of LSCs, promoting their proliferation and migration [105]. Wang et al. also found that METTL3, which is highly expressed in the herpes stromal keratitis (HSK) mouse model, promotes pathological angiogenesis through canonical Wnt and VEGF signaling in vitro and in vivo, providing a potential pharmacological target to prevent the progression of corneal neovascularization in HSK [106].

Furthermore, Shan et al. found that FTO silencing inhibited endothelial cell function and corneal neovascularization by increasing m6A modification levels, which prompted YTHDF2 to degrade FAK mRNA, resulting in decreased Focal Adhesion Kinase (FAK) protein expression [83].

In summary, m6A modification plays a pivotal role in the formation of corneal neovascularization and injury repair by regulating key genes and signaling pathways. Therefore, precise modulation of m6A modification levels may provide novel strategies for the prevention and treatment of corneal neovascularization.

#### Cataract

The lens focuses light onto the retina through its transparent biconvex structure, forming a clear image essential for visual function [107]. Cataracts are caused by lens opacities, most of which develop after birth, primarily due to aging and oxidative stress [108]. Cataracts are classified into age-related cataract (ARC), congenital cataract, traumatic cataract, and metabolic cataract. ARC primarily affects individuals over 50 years of age and results from the gradual opacification of the lens with age, making it the most common type of cataract. Based on anatomical location or opacity features, cataracts can also be categorized as nuclear (opacification of the fetal and adult nucleus), cortical (spoke-like opacification of the lens fibers), and posterior (plaque-like opacification in the back portion of the lens fibers). From 2000 to 2020, cataract-related blindness increased by 30%, while moderate to severe visual impairment rose by 93%. Although advances in cataract surgery have reduced cataract-related blindness, the overall number of cataract cases continues to rise due to the aging population, particularly the increasing number of individuals over 60 years of age [108].

In the Malay adult population in Singapore, Lim et al. found through Mendelian randomization that rs9939609 in the FTO gene was significantly associated with an increased risk of nuclear cataract, but not cortical or posterior subcapsular cataract [109]. In a northern Indian population, Chandra et al. conducted a case-control study and found that this locus was significantly associated with an increased risk of cataracts, although it did not clearly differentiate between cataract subtypes [110]. Despite differences in study methods, these findings suggest that FTO gene polymorphisms may influence the development of cataracts through mechanisms independent of obesity. However, the specific mechanisms of action and affected cataract subtypes appear to vary across populations and warrant further investigation.

Investigating the effect of lens epithelium cell (LEC) RNA transcripts on cataract formation is critical, as the integrity and metabolic activity of LECs are essential for maintaining lens transparency. These functions depend on a normal transcriptome. Multiple proteins involved in m6A modification have been found to be aberrantly expressed in LECs from cataract patients. For instance, levels of m6A-modified circular RNAs (circRNAs) were found to be significantly reduced in LECs from age-related cataract (ARC) patients compared to normal controls, with high m6A-modified circRNAs predominantly downregulated. These m6A-modified circRNAs are closely associated with ARC-related pathways, including oxidative stress, DNA damage repair, and autophagy, suggesting that m6A modification may play a role in ARC development by inhibiting circRNA expression. Additionally, the demethylase ALKBH5 is significantly upregulated in ARC patients and may affect m6A-modified circRNAs through demethylation, thereby promoting ARC pathogenesis [111].

Additionally, Li et al. determined that m6A modification was significantly upregulated in ARC through MeRIP-Seq, with methyltransferase METTL3 being significantly increased in ARC tissues and high glucose-induced lens epithelial cells (HLE-B3). METTL3 overexpression was shown to promote m6A modification and the expression of hascirc0007905, which regulates EIF4EBP1 expression by sponging miR-6749-3p. Upregulation of EIF4EBP1 suppressed proliferation and promoted apoptosis in HLE-B3 cells, driving ARC development. This study highlights the critical role of the METTL3/hascirc0007905/miR-6749-3p/EIF4EBP1 axis in ARC and provides a novel target for ARC treatment [112].

Diabetic cataract (DC) is a type of metabolic cataract caused by lens metabolic disorders resulting from long-term hyperglycemia (HG), leading to lens opacification. Its pathogenesis is closely associated with oxidative stress and apoptosis in lens epithelial cells (LECs). Recent studies have revealed that m6A modification reduces the expression of its target gene superoxide dismutase 2 (SOD2) through METTL3-mediated miR-4654 maturation, exacerbating oxidative stress and apoptosis in LECs, and promoting lens opacification [113]. Moreover, METTL3 was found to be upregulated in high glucose-induced HLECs by MeRIP-Seq analysis, where it contributes to the pathogenesis of DC by targeting the 3’ UTR of ICAM-1, stabilizing its mRNA, and promoting protein expression [114].

Interestingly, Cai et al. revealed a significant increase in m6A modification levels in DCs and notable changes in m6A modification across multiple mRNAs, as determined through microarray analysis of the m6A transcriptome. Gene ontology (GO) and pathway enrichment analysis indicated that these differentially expressed mRNAs were primarily enriched in the ferroptosis pathway, suggesting that m6A modification may play a role in DC progression by regulating this pathway. Furthermore, the RNA methyltransferase RBM15 was found to be significantly upregulated in DCs, potentially promoting oxidative stress and ferroptosis in lens epithelial cells through m6A modification, thereby driving disease progression [115].

Overall, while significant progress has been made in studies of m6A modification in cataracts, a consistent observation across different types of cataracts is that METTL3 promotes LEC apoptosis. However, many unresolved questions remain in the existing literature. For example, is the regulatory mechanism of m6A modification in DC consistent with that in ARC? Is there a specific m6A modification pattern or target that can distinguish DC from ARC? Furthermore, the precise role of the ferroptosis pathway in DC remains unclear. How does m6A modification affect the survival of LECs by regulating the ferroptosis pathway, thereby influencing DC pathogenesis? Addressing these questions will help further elucidate the complex mechanisms of m6A modification in cataracts and provide new insights for the development of targeted therapeutic strategies.

#### Glaucoma

Glaucoma is a leading irreversible cause of blindness worldwide, characterized by optic nerve damage and the progressive loss of retinal ganglion cells (RGCs). The primary causes are elevated intraocular pressure (IOP) or impaired retinal blood flow, which result in thinning of the optic nerve fiber layer and cupping of the optic disc, leading to visual field defects and decreased visual acuity [116]. However, not all patients with elevated IOP develop glaucoma, and some individuals may present with typical glaucomatous optic neuropathy, known as normal tension glaucoma (NTG), even within the normal IOP range. The pathogenesis of glaucoma is multifactorial, involving genetics, oxidative stress, mechanical compression, vascular compression, neuroinflammation, and mitochondrial dysfunction. Oxidative stress is closely associated with cellular aging, mitochondrial dysfunction, and neuroinflammation, all of which contribute to the pathological progression of glaucoma [117]. With the aging global population, the incidence of glaucoma is expected to continue rising, presenting a significant public health challenge [118].

Pseudoexfoliation glaucoma (PXG) is a major form of secondary glaucoma, characterized by the accumulation of abnormal extracellular fibrillar material in anterior segment structures. This leads to increased trabecular meshwork outflow resistance, elevated IOP, and, ultimately, optic nerve injury and visual field defects [119, 120].

Guan et al. analyzed the m6A modification profile in aqueous humor (AH) from PXG patients and found that m6A levels were significantly higher than those in ARC patients, suggesting that m6A modification may play an important regulatory role in PXG. Transcriptome analysis revealed numerous differential m6A modification peaks in the AH of PXG patients, primarily enriched in coding sequences (CDS) and 3’-untranslated regions (3’-UTR), which may influence the pathological process of PXG by regulating mRNA stability, translational efficiency, and intracellular localization. Additionally, m6A modification is closely associated with extracellular matrix (ECM) formation and histone deacetylation in PXG. Genes such as MMP14, ADAMTSL1, FN1, and HDAC1 exhibited significant m6A methylation and expression changes in PXG, highlighting the importance of ECM remodeling and cell phenotype regulation in the disease [121].

Additionally, recent studies have shown that lncRNAs in the aqueous humor (AH) of PXG patients exhibit significant m6A modification differences, which may contribute to the pathogenesis of PXG by influencing the expression and function of lncRNAs. By constructing an lncRNA-miRNA-mRNA competitive endogenous RNA (ceRNA) network, it was found that specific lncRNAs (e.g., ENST00000485383) may bind multiple miRNAs through a sponge mechanism, relieving their inhibition of downstream mRNA (e.g., ROCK1) expression. This interaction may influence the inflammatory response of retinal pigment epithelial cells and the fibroproliferation of trabecular meshwork cells [122].

These findings not only offer new insights into the pathogenesis of PXG but also provide a direction for further investigation into its pathophysiology and potential therapeutic targets, which could lead to breakthroughs in PXG research. Future studies should validate these mechanisms through cellular and animal models and assess their generalizability across diverse ethnic and geographical populations.

The degeneration of RGCs shares a similar pathomechanism with Retinal Ischemia-Reperfusion (RIR) injury [123]. Recent studies have shown that RIR injury activates the autophagic process, leading to RGC death and impaired retinal electrophysiological function. Further mechanistic studies revealed that METTL3-mediated m6A modification levels decreased after RIR injury. Overexpression of METTL3 reduced FoxO1 protein expression by increasing m6A modification on FoxO1 mRNA, thereby inhibiting autophagy activation and protecting RGCs. Moreover, the use of FoxO1 inhibitors also alleviated RGC loss and retinal dysfunction caused by RIR injury by inhibiting autophagy [124]. In contrast, YTHDF2 is highly expressed in mouse RGCs, and its conditional knockout (cKO) significantly increased dendritic branch complexity in RGCs, while improving visual function. In an acute intraocular pressure elevation (AOH)-induced glaucoma model, RGCs from YTHDF2 cKO mice showed greater resistance to injury, reduced dendritic atrophy, and less neuronal loss [125].

Glaucoma Filtration Surgery (GFS) is a standard surgical approach for treating glaucoma, but postoperative scar formation remains the primary cause of surgical failure [126]. Studies have shown that excessive activation of human Tenon’s capsule fibroblasts (HTFs) and abnormal accumulation of extracellular matrix (ECM) are key factors in scar formation [127]. TGF-β1 was found to significantly enhance the proliferation and ECM accumulation of HTFs, elevating m6A modification levels by upregulating METTL3. Moreover, METTL3 modulated HTF function by regulating mothers against decapentaplegic homolog 3 (Smad3) expression. Overexpression of Smad3 reversed the inhibitory effect of METTL3 inhibition on ECM accumulation and cell proliferation. In a rabbit GFS model, METTL3 and Smad3 expression were significantly elevated in postoperative scar tissue, further confirming the critical role of the METTL3/Smad3 axis in scar formation after GFS [128].

In summary, m6A modification could serve as a potential therapeutic target for glaucoma by influencing gene expression and regulating cell phenotype. However, current studies have not fully elucidated the universal role of m6A modification in different glaucoma subtypes or its specific molecular mechanisms. While certain m6A-related genes, such as METTL3 and YTHDF2, have been identified as regulated in glaucoma models, it remains unclear whether the m6A modification pattern is consistent across all patients or universally applicable across different ethnic groups and individuals. This question requires further in-depth investigation. Therefore, accurately regulating m6A modification may represent a crucial direction for future glaucoma treatment research.

#### Uveitis

Uveitis is a complex disease characterized by intraocular inflammation, primarily affecting uveal structures such as the iris, ciliary body, and choroid. It is classified into two major categories: infectious and non-infectious. The former is primarily caused by pathogens, including bacteria, viruses, or parasites, while the latter is often associated with systemic immune-mediated inflammatory diseases, such as juvenile idiopathic arthritis (JIA), ankylosing spondylitis (AS), and Behcet’s disease [129]. Uveitis can present as either acute or chronic inflammation, involving the anterior, middle, or posterior segments of the eye, or even the entire uvea. Symptoms vary and include eye redness, pain, photophobia, blurred vision, and dark shadows, which, in severe cases, can lead to vision loss or blindness [130]. Globally, uveitis is a significant cause of visual impairment, particularly among children and adolescents, with a high incidence and rate of blindness. Due to its complex etiology, diagnosis, and treatment require close collaboration between ophthalmology and rheumatology is required to identify the underlying cause and develop an individualized treatment plan [131].

Microglia are the primary immune effector cells of the retina, exhibiting high plasticity and playing a critical role in the pathogenesis of uveitis. Their activation state is primarily categorized into two types: the M1 pro-inflammatory “classical activation” state and the M2 anti-inflammatory “alternative activation” state [132].

In the experimental autoimmune uveitis (EAU) mouse model and a LPS/IFN-γ-induced inflammatory environment, He et al. observed downregulation of FTO expression. FTO deletion significantly enhanced the pro-inflammatory properties of microglia, as evidenced by increased secretion of inflammatory factors, enhanced cell migration, and significantly elevated chemotaxis toward CD4 T cells. RNA sequencing analysis identified GPC4 as a downstream target gene of FTO, with its upregulation closely associated with the downregulation of YTHDF3. Increased GPC4 expression further activated the TLR4/NF-κB signaling pathway, driving microglial polarization toward the M1 phenotype and exacerbating the inflammatory response. In vivo, FB23-2, an FTO inhibitor, significantly aggravated inflammation in EAU by activating the GPC4/TLR4/NF-κB signaling axis. However, this exacerbated inflammation was alleviated by TAK-242, a TLR4 inhibitor [89].

Similarly, FTO expression in retinal pigment epithelial (RPE) cells was significantly downregulated in the EAU mouse model. FTO loss significantly enhanced the pro-inflammatory properties of RPE cells, as evidenced by increased secretion of inflammatory factors, enhanced cell migration, and decreased expression of tight junction proteins (e.g., ZO-1 and occludin). Further studies revealed that FTO regulates the translation efficiency of ATF4 by modulating its m6A modification level. Knockdown of FTO resulted in increased m6A modification of ATF4, thereby suppressing ATF4 protein expression. As a key transcription factor, reduced ATF4 expression activates the p-STAT3 signaling pathway, promoting the secretion of inflammatory factors and the degradation of tight junction proteins. Moreover, low FTO expression has been associated with enhanced proliferative capacity in RPE cells, potentially exacerbating the inflammatory response [133].

Moreover, significant downregulation of YTHDC1 expression in retinal microglia is closely associated with uveitis. Loss of YTHDC1 impairs the maintenance of SIRT1 mRNA stability, making it more prone to degradation, which in turn reduces SIRT1 protein levels. This alteration promotes acetylation and phosphorylation of STAT3, ultimately driving microglial polarization toward the M1 phenotype. This polarization is characterized by a marked upregulation of pro-inflammatory markers such as iNOS and COX2, along with increased expression of inflammatory factors like TNF-α, thereby exacerbating the inflammatory response in uveitis [134].

Pathogenic Th17 cells play a critical role in the pathology of uveitis [135]. The significance of METTL3 in EAU was first demonstrated in 2023 by Zhao et al. It was observed that METTL3 expression and m6A levels were both reduced in the ocular tissues and T cells of EAU mice. Lentivirus-mediated overexpression of METTL3 restored m6A levels, significantly alleviated EAU symptoms, reduced inflammatory cell infiltration, and improved retinal structural damage. METTL3 decreases Th17 cell activity by stabilizing ASH1L mRNA, inhibiting pathogenic Th17 cell responses, and reducing the expression of IL-17 and IL-23R. YTHDC2 plays a pivotal role in regulating ASH1L mRNA stability through METTL3, and its knockdown weakens the regulatory effect of METTL3 [91].

In the same year, Wei et al. injected miR-338-3p overexpressing dendritic cells (DCs) into mice with EAU and observed more severe symptoms, including increased inflammatory cell infiltration and retinopathy, as observed through ophthalmoscopy and OCT imaging. DCs are central to the development of Th17 cells, acting as a crucial bridge between innate and adaptive immunity. They provide essential microenvironmental support for Th17 cell polarization by secreting pro-inflammatory cytokines such as IL-6, IL-1β, and IL-23. METTL3 significantly upregulated miR-338-3p expression by promoting the maturation of pri-miR-338 and alleviating its inhibition of the p38 signaling pathway by targeting dual-specificity phosphatase 16 (Dusp16), thereby enhancing the ability of DCs to secrete cytokines associated with Th17 cell polarization [136].

In summary, the effect of METTL3 in uveitis varies depending on cell type and experimental conditions. In the EAU model, METTL3 expression was significantly reduced in T cells and ocular tissues, and this reduction was closely associated with disease severity, suggesting that downregulation of METTL3 may promote the pathogenic response of Th17 cells, thereby exacerbating the development of EAU. However, METTL3 expression in DCs exhibited the opposite trend, ultimately enhancing Th17 cell polarization and pathogenic responses. This difference highlights the complex regulatory mechanisms of METTL3 in various cell types and pathological conditions, underscoring its versatility in immune responses. Therefore, differences in cell types and experimental conditions must be thoroughly considered when investigating METTL3’s role in uveitis to gain a comprehensive understanding of its mechanism in disease development.

Zhou et al. were the first to elucidate the distinctive metabolic profile of modified nucleosides in the serum of uveitis patients. Using liquid chromatography-tandem mass spectrometry (LC-MS/MS), they analyzed a total of 23 modified nucleosides, identifying 13 that were significantly altered in patients compared to healthy controls. Notably, different subtypes of uveitis exhibited unique combinations of these modified nucleosides. This discovery not only introduces novel biomarkers for more accurate diagnosis but also offers fresh insights into the underlying pathomechanisms of uveitis [137]. Furthermore, the findings underscore the potential role of RNA modifications in uveitis, laying a theoretical foundation for the development of “RNA epigenetics-based” diagnostic and therapeutic strategies. Such strategies could significantly advance the progress of precision medicine for uveitis.

#### Graves’ ophthalmopathy (GO)/thyroid eye disease (TED)

GO, also known as TED, is the most common extrathyroidal manifestation of Graves’ disease and is classified as an autoimmune disorder. Its pathogenesis is complex and primarily involves the abnormal expression of thyroid-stimulating hormone receptor (TSHR) in orbital tissues, which triggers inflammation and tissue remodeling in orbital fat, fibrocytes, and extraocular muscles (EOMs). This leads to exophthalmos, eyelid retraction, diplopia, and visual impairment. The pathogenesis of GO is closely associated with genetic and environmental factors, as well as thyroid dysfunction [138]. Treatment for GO depends on the severity and activity of the disease. Mild GO is generally managed symptomatically, while glucocorticoids are commonly required for moderate to severe active GO, although the recurrence rate remains high. Recently, targeted therapies such as teprotumumab, an insulin-like growth factor 1 receptor (IGF-1R) blocker, have shown promising efficacy, offering new hope for treatment. Surgical intervention may be necessary for refractory or advanced cases of GO [139, 140].

Zhu et al. found significantly elevated m6A levels in EOM samples from GO patients, suggesting that m6A methylation may play a crucial role in the pathology of GO. Aberrant expression of several m6A methylation regulators, including WTAP, ALKBH5, and YTHDF2, was also observed. Changes in the expression of these regulators were closely associated with the upregulation of genes involved in inflammation and immune responses, indicating that m6A methylation may influence the inflammatory response in GO by regulating these genes. Furthermore, the expression of inflammatory cytokines, such as IL-6, TNF, and IFN-γ, was significantly increased in the EOM of GO patients, further supporting the critical role of m6A methylation in the inflammatory pathology of GO [141]. Importantly, specific autoantibodies against EOM antigens have been identified in GO patients, strongly suggesting that EOM is a major target of autoimmune attack [142, 143].

DR is closely associated with the presence of DM, and similarly, the onset of GO/TED is strongly linked to thyroid disease. Recent studies have demonstrated a clear association between m6A modification and thyroid disease, which not only aids in understanding the pathogenesis of GO/TED but also provides a theoretical foundation for the development of targeted treatments and preventive strategies. HNRNPC significantly enhanced the transcriptional activity and translational efficiency of ATF4 by mediating its m6A modification. Activated ATF4 induced apoptosis and necrosis of thyroid follicular epithelial cells (ThyFoEp) through the ER stress pathway, contributing to the progression of Hashimoto’s thyroiditis (HT) [144]. Additionally, METTL3 expression was downregulated in thyroid tissue from Graves’ disease (GD) patients, accompanied by upregulation of suppressor of cytokine signaling (SOCS) family members. This suggests that METTL3 may influence immune cell function by regulating the m6A modification of SOCS family mRNAs, thereby playing a role in the pathogenesis of GD [145].

Despite significant progress in revealing epigenetic regulatory networks in thyroid-associated ophthalmopathy (TAO), this study has some limitations, including a small sample size, a lack of mechanistic validation, insufficient investigation of dynamic changes in epigenetic modifications, and limited depth of multi-omics data integration. Such limitations may impact the scope and practical value of the study’s conclusions. Future studies should validate these findings further and explore their potential clinical applications in TAO management by expanding sample size, conducting in vitro and in vivo experiments, analyzing dynamic changes in epigenetic modifications, and integrating multi-omics data more comprehensively.

Recent studies have revealed the epigenetic regulatory network in thyroid-associated ophthalmopathy (TAO) through the integration of multi-omics sequencing data, including DNA methylation, RNA-seq, and tRFs sequencing. These studies have shown that epigenetic modification levels undergo significant changes in TAO patients and are closely associated with the PI3K-Akt and IL-17 signaling pathways. In particular, m6A methylation regulates numerous genes involved in key processes such as cytokine production, immune response, and cell chemotaxis [146]. However, the study relied solely on bioinformatics analysis. Moving forward, dynamic changes in epigenetic modifications should be analyzed in greater detail through in vitro and in vivo experiments. Additionally, the integration of multi-omics data should be further refined to validate these findings and explore their potential applications in the clinical management of TAO.

Although these studies provide new insights into the pathogenesis of GO/TED, existing research is limited to a single molecular mechanism and does not fully elucidate the complex interactions between m6A methylation and other epigenetic modifications. An important question for future studies is whether they can explore the synergistic effects of various epigenetic modifications (e.g., DNA methylation, non-coding RNAs) in GO/TED, thereby offering a more comprehensive theoretical foundation for precision treatment.

### Posterior segment diseases

#### Diabetic retinopathy (DR)

Diabetic retinopathy (DR) is one of the primary microvascular complications of diabetes mellitus (DM) and remains the leading cause of vision loss in working-age individuals. According to the 2021 data from the International Diabetes Federation (IDF), approximately 537 million adults worldwide are living with DM, with projections indicating that this number will rise to 783 million by 2045 [147]. It is estimated that between 20% and 50% of DM patients will develop DR, a condition closely associated with prolonged hyperglycemia (HG), hypertension, and obesity [148]. Children and adolescents diagnosed with type 2 diabetes (T2D) face an extended disease course and are at a heightened risk for DR, with a significantly increased prevalence observed beyond five years after diagnosis [149]. Current treatment options for DR include anti-VEGF therapy, laser photocoagulation, and vitrectomy, although these interventions have limitations in terms of efficacy and are often accompanied by side effects [150]. Consequently, annual DR screening is strongly recommended for all patients with DM. Concurrently, further investigations into the pathogenesis of DR, as well as the identification of novel diagnostic biomarkers and therapeutic targets, are essential for improving patient outcomes and reducing the risk of vision impairment. Recent advances in research on m6A modification—an important epigenetic modification—have shed new light on its potential role in DR, offering valuable insights into the complex mechanisms underlying this disease.

The Neurovascular Unit (NVU) encompasses a variety of cellular components, including retinal vascular endothelial cells, pericytes, neuroglial cells (such as Müller cells and retinal microvascular cells, rMC), neurons, and resident immune cells (e.g., microglia). The primary functions of the NVU are to maintain the integrity of the blood-retinal barrier (BRB), regulate retinal blood flow, support neural function, and modulate immune responses, thereby ensuring the normal physiological activity of the retina [151]. Diabetic retinopathy (DR) is classified into two main types: non-proliferative diabetic retinopathy (NPDR) and proliferative diabetic retinopathy (PDR) [152]. NPDR is further divided into mild, moderate, and severe stages based on the severity of the disease. It is primarily characterized by microaneurysms, hemorrhages, and exudates, which reflect abnormal changes in the retinal vasculature. In contrast, PDR represents the advanced stage of DR, typically triggered by retinal ischemia and hypoxia, which induce retinal neovascularization (RNV). These neovascularizations are fragile and prone to rupture and bleeding, potentially leading to significant vision loss and, in severe cases, blindness [153].

Studies have shown that abnormal changes in the migration, proliferation, and capillary lumen formation of retinal microvascular endothelial cells (RMECs) are closely associated with the development of retinal neovascularization (RNV) and the progression of proliferative diabetic retinopathy (PDR) [154]. Additionally, retinal microglial cells (rMCs), the principal glial cells of the retina, undergo gliosis under pathological conditions and secrete various pro-inflammatory factors, including IL-1β, IL-6, TNF-α, and VEGF. These factors play a critical role in key pathological processes such as retinal inflammation, vascular leakage, and abnormal RNV formation [155, 156]. Lysine acetyltransferase 1 (KAT1), a key epigenetic regulator, has garnered attention due to its potential involvement in DR. Research by Qi et al. revealed that KAT1 expression is significantly downregulated in the retinal tissue of diabetic mice. Interestingly, overexpression of KAT1 was found to notably inhibit retinal inflammation, RNV formation, and vascular leakage. Molecular mechanistic studies indicated that KAT1 accelerates the degradation of ITGB1 mRNA and suppresses its protein expression by activating the transcription of YTHDF2, which enhances the m6A modification of ITGB1 mRNA. This process not only impedes the proliferation and migration of RMECs but also mitigates the abnormal activation and inflammatory response of rMCs by inhibiting the FAK/PI3K/AKT signaling pathway [157]

Pericytes, which surround the endothelial cells (ECs) of capillaries, play a crucial role in maintaining vascular integrity and function [158, 159]. Dysfunction of pericytes can lead to endothelial cell abnormalities and contribute to microvascular complications [160]. In diabetic retinopathy (DR), hyperglycemia (HG) triggers pericyte dysfunction, resulting in microaneurysms, destruction of the blood-retinal barrier (BRB), disruption of pericyte-endothelial cell interactions, and the formation of pathological retinal neovascularization (RNV). These alterations are closely associated with capillary remodeling, fibrosis, and retinal detachment [161, 162]. In the context of diabetes mellitus (DM), the upregulation of m6A modification mediated by METTL3 suppresses the expression of key genes, including PKC-η, FAT4, and PDGFRA. This inhibition impairs the survival, proliferation, and differentiation of pericytes, ultimately contributing to retinal vascular complications. In vitro experiments demonstrated that silencing METTL3 significantly alleviated high glucose-induced apoptosis and dysfunction of pericytes. Additionally, specific knockdown of METTL3 in pericytes in a diabetic mouse model reduced pericyte loss, diminished retinal vascular leakage, and alleviated microangiopathy [163].

The retinal pigment epithelium (RPE) normally maintains BRB integrity, regulates nutrient and ion transport, absorbs light energy, and engulfs the outer photoreceptor segment, while also secreting multiple factors to maintain retinal homeostasis [164]. Recently, pyroptosis, a form of programmed cell death, has garnered significant attention due to its crucial role in inflammation and cell injury [165]. In DR, high glucose-induced RPE dysfunction is a critical pathological event. High glucose has been shown to inhibit RPE cell proliferation and induce apoptosis. For example, METTL3 overexpression targets PTEN and activates the Akt signaling pathway by upregulating miR-25-3p, thereby alleviating the inhibitory effect of high glucose on RPE cell proliferation and reducing both apoptosis and pyroptosis [166]. Similarly, circFAT1 is significantly downregulated in high glucose-induced RPE, while its overexpression protects cells from high glucose-induced injury by promoting autophagy and inhibiting pyroptosis [167]. Furthermore, downregulation of miR-192 expression promotes FTO expression and activation of NOD-like receptor pyrin domain containing 3 (NLRP3) inflammasomes under high glucose (HG) conditions, subsequently promoting pyroptosis of RPE cells [168].

Moreover, in DR, pathological factors such as high glucose (HG) not only inhibit RPE cell proliferation and induce apoptosis but also disrupt the balance of secreted factors, including the upregulation of pro-angiogenic factors (e.g., VEGF), the downregulation of anti-angiogenic factors (e.g., PEDF), and the increase of inflammatory factors (e.g., IL-6, IL-8). These changes lead to BRB destruction, vascular leakage, pathological RNV formation, and promote DR progression [164]. Therefore, targeting these molecular mechanisms, beginning with RPE cells, could offer new strategies for treating DR and may delay disease progression by regulating pyroptosis and secretory function.

The phenotypic switch of cells is a pivotal biological event, with endothelial-mesenchymal transition (EndoMT) referring to the loss of endothelial cell characteristics and the acquisition of mesenchymal traits by endothelial cells. This process is intricately linked to embryonic development, tissue repair, and several pathological conditions [169]. High glucose impairs endothelial cell (EC) function and induces EndoMT [170]. EndoMT is considered a critical early event in the pathological angiogenesis of retinal vasculopathy, and its regulation by m6A modification plays a crucial role in diabetic retinopathy (DR). METTL3 has been shown to inhibit EndoMT by enhancing the stability of SNHG7 and reducing the stability of MKL1 mRNA. In animal models, overexpression of METTL3 improved retinal architecture and reduced the expression of EndoMT markers, while knockdown of SNHG7 partially reversed these effects. These findings suggest that METTL3 plays a protective role in DR and highlight potential therapeutic strategies targeting m6A modification and non-coding RNA regulation [171].

The regulation of FTO plays a critical role in the progression of diabetic retinopathy (DR). Studies have demonstrated that FTO expression is upregulated in both diabetic patients and animal models, leading to a reduction in m6A modification levels. FTO decreases the stability of TNIP1 mRNA, thereby downregulating its protein expression, activating the NFκB pathway, and increasing inflammatory factor levels, such as IL-1β and IL-18. These changes exacerbate diabetes-induced retinal vascular leakage and acellular capillary formation. Endothelial cell-specific FTO knockout mice exhibit protection against DR, and sustained expression of TNIP1 through AAV-mediated gene therapy significantly mitigates diabetes-induced endothelial dysfunction [88]. In addition, FTO promotes angiogenesis by regulating the stability of CDK2 mRNA, facilitating endothelial cell cycle progression and tip cell formation. FTO also contributes to microvascular leakage, retinal inflammation, and neurodegenerative changes in DR by disrupting the interactions between endothelial cells, pericytes, and microglia. Mechanistically, the upregulation of FTO is driven by lactate-mediated histone lactylation (H3K18la), and its demethylase activity impacts CDK2 mRNA stability in a YTHDF2-dependent manner. The FTO inhibitor FB23-2 and its nanoplatform, NP-FB23-2, have been developed and shown to possess effective targeting and therapeutic effects in mouse models [90].

Inflammation plays a central role in the development and progression of diabetic vasculopathy. Notably, the polarization of macrophages into either the M1 pro-inflammatory phenotype or the M2 anti-inflammatory phenotype significantly influences the inflammatory response [172]. Downregulation of FTO expression is strongly associated with macrophage polarization toward the M1 phenotype under diabetic conditions. Recent studies have shown that FTO regulates mRNA stability through an m6A-YTHDF2-dependent pathway, which subsequently affects macrophage polarization. Knockdown of FTO accelerates the degradation of STAT1 and PPAR-γ mRNA, promoting M1 polarization. In contrast, silencing YTHDF2 increases the stability and expression of these mRNAs, inhibits M1 polarization, and reduces the inflammatory response.

Moreover, FTO modulates inflammation by stabilizing FGF2 mRNA and activating the PI3K/AKT signaling pathway. Since fibroblast growth factor 2 (FGF2) promotes macrophage polarization toward the M2 phenotype, the downregulation of FTO, resulting in decreased FGF2 expression, exacerbates M1 polarization and further intensifies inflammation [86].

In summary, the expression levels of FTO in DR studies show significant variability across different cell types and pathological conditions. In fibrovascular membranes (FVMs) from patients with PDR, FTO expression is upregulated and closely associated with elevated inflammatory factors, vascular leakage, and acellular capillary formation. This suggests that the upregulation of FTO in retinal endothelial cells and pericytes may exacerbate diabetes-induced vasculopathy. Conversely, FTO expression is significantly downregulated in THP-1 cells (mimetic macrophages) cultured under HG conditions, which correlates with macrophage polarization toward the pro-inflammatory M1 phenotype, thus intensifying the inflammatory response. This contrast underscores the complex regulation of FTO across various cell types and highlights its involvement in multiple pathways within the pathogenesis of DR. Therefore, when considering FTO as a potential therapeutic target, it is essential to carefully assess its specific role in distinct cell types and pathological contexts.

M2 macrophages are typically considered anti-inflammatory and play key roles in tissue repair, immune regulation, and the resolution of inflammation under normal physiological conditions. However, under certain pathological states, the function of M2 macrophages may shift, potentially exacerbating the pathological process. For instance, Gao et al. demonstrated that after corneal injury, a substantial release of cytokines and chemokines induces macrophage polarization toward the M2 phenotype. Nevertheless, this polarization can alter M2 macrophage function, thereby enhancing their role in promoting angiogenesis [173]. In contrast, under diabetic conditions, macrophages tend to polarize toward the pro-inflammatory M1 phenotype, a shift closely associated with aggravated tissue inflammation and vascular dysfunction [174, 175].

The rs9939609 polymorphism in the FTO gene is strongly associated with an increased risk of diabetic retinopathy (DR) in patients with type 1 diabetes (T1D). T1D patients carrying the AA genotype exhibit a significantly higher risk of developing DR (OR = 2.203, p = 0.008), whereas the AT genotype appears to confer a protective effect (OR = 0.433, p = 0.003). This association may stem from the regulation of inflammatory status and lipid metabolism by the FTO gene. Specifically, patients with the AA genotype show elevated levels of inflammatory markers, such as CRP, IL-6, and ICAM-1, suggesting that chronic low-grade inflammation may play a pivotal role in the progression of DR. Additionally, the influence of FTO gene polymorphisms on lipid profiles provides a metabolic basis for the increased risk of DR. These findings highlight the critical role of the FTO gene in T1D-related retinopathy and suggest potential new targets for therapeutic intervention in T1D complications [176].

Microglia are resident immune cells in the retina that play a crucial role in retinal inflammation [177]. Under HG conditions, retinal microglia exhibit reduced expression of tumor necrosis factor alpha-induced protein 3 (TNFAIP3, A20), leading to a shift toward increased pro-inflammatory polarization. Moreover, decreased levels of ALKBH5 enhance m6A modification, which accelerates the degradation of A20 mRNA [178]. Additionally, a reduction in ALKBH5 promotes the expression of WNT5A, thereby accelerating angiogenesis in ischemic tissue. [79]. However, the specific mechanisms underlying these processes in the retina remain poorly understood and require further investigation.

DR is a secondary complication of DM, with DM serving as a prerequisite for the development of DR, suggesting a potential link between the two. Since 2015, studies have increasingly shown that reduced levels of m6A modification are strongly associated with the onset of T2D, positioning m6A modification as a potential risk factor and biomarker for the disease [179]. Understanding the regulatory mechanisms of m6A modification in the development of DM could lead to the identification of novel therapeutic targets. Such insights could pave the way for more precise treatments, reduce the incidence of DM and its complications, improve patients’ quality of life, and alleviate the societal medical burden.

#### Retinopathy of prematurity (ROP)

ROP is a prevalent retinal vascular disorder in premature infants, particularly affecting those with very low birth weight and low gestational age. It is a leading cause of visual impairment and blindness in children. The pathogenesis of ROP is complex, involving multiple factors such as premature birth, oxygen exposure, infection, inflammation, and genetic susceptibility [180]. The pathological progression of ROP occurs in two stages: the initial abnormal proliferation of retinal vessels, which can potentially lead to retinal detachment, followed by abnormal vessel dilation and fibrosis, further impairing visual acuity [181]. In recent years, the incidence and severity of ROP have increased, largely due to the improved survival rates of preterm infants, particularly in low- and middle-income countries. Currently, laser photocoagulation and anti-VEGF drug injections are the primary treatments; however, these approaches have limitations, including visual field defects, myopia, and systemic side effects. Thus, understanding the pathomechanisms of ROP and developing new diagnostic markers and treatment strategies are crucial for improving the visual outcomes of premature infants [182]

Zhou et al. reported significant changes in the m6A modification levels of 88 circRNAs in oxygen-induced retinopathy (OIR) mouse models, with 56 circRNAs exhibiting hypermethylation and 32 showing hypomethylation. Gene ontology (GO) and Kyoto Encyclopedia of Genes and Genomes (KEGG) analyses revealed that the host genes of these circRNAs were primarily involved in pathways related to cellular processes, protein binding, and lysine degradation. Additionally, circRNA-miRNA-mRNA network analysis further suggested that m6A-modified circRNAs may regulate retinopathy of prematurity (ROP) through the competing endogenous RNA (ceRNA) mechanism [183].

In a similar OIR mouse model, Peng et al. observed significant alterations in m6A modification levels in 1321 mRNAs and 192 lncRNAs in retinal tissue, the majority of which exhibited hypomethylation. GO analysis revealed that hypermethylated mRNAs were predominantly involved in multicellular biological processes and organelle functions, whereas hypomethylated mRNAs were associated with cellular metabolism and binding functions. KEGG analysis indicated that hypermethylated mRNAs were linked to the PI3K-Akt signaling pathway, while hypomethylated mRNAs were related to autophagy and ubiquitin-mediated proteolysis. These findings underscore the pivotal role of m6A modification in retinal neovascularization and provide a theoretical foundation for the development of novel therapeutic targets for ROP [184].

Pathological angiogenesis, triggered by retinal hypoxia, is a key component of ROP pathology [185]. In response to hypoxic conditions, m6A modification levels were notably increased in both HU-VEC cells and mouse retinas. This elevation was predominantly mediated by METTL3, which regulated the Wnt signaling pathway. The influence of METTL3 on this pathway led to the altered expression of critical genes, such as LRP6 and DVL1, ultimately driving the progression of pathological angiogenesis [80].

Furthermore, oxidative stress, pyroptosis, and autophagy dysfunction have been shown to interact in the pathophysiology of retinal neovascularization in OIR mice, establishing a vicious cycle. Therefore, targeting oxidative stress, inhibiting pyroptosis, and restoring autophagic function could provide novel strategies for preventing and treating retinal neovascularization [186].

Notably, the physiological and pathological conditions of preterm infants differ significantly from those of adult animals, as the retinas of preterm infants are immature and more vulnerable to hypoxia and oxygen exposure. While many current studies rely on animal models, such as the OIR mouse model, which replicate certain pathological features, these models fail to fully capture the complexity and individual variability of ROP in humans. As a result, the applicability of findings from animal models to preterm infants may be limited. Future research is crucial to further investigate the relevance of these findings to preterm infants and to develop diagnostic markers and treatment strategies specifically tailored for this population, ultimately providing new approaches for diagnosing and treating ROP.

#### Retinitis pigmentosa (RP)

RP is an inherited retinal degenerative disorder affecting approximately 2.5 million people worldwide. It is characterized by the progressive degeneration of rods and cones, leading to night blindness, loss of peripheral vision, and ultimately, total blindness [187]. The disorder can be inherited through various patterns, including autosomal dominant, autosomal recessive, X-linked, and undefined modes of inheritance. Mutations in genes such as RHO, USH2A, and RPGR have been implicated in the disease. In recent years, emerging therapies, including gene therapy, cell transplantation, and retinal prostheses, have made significant advances. Luxturna, a gene therapy targeting RPE65 mutations, has shown considerable efficacy in clinical trials. However, challenges persist in the treatment of RP, including the genetic heterogeneity of mutations, limitations in targeting specific mutations, and the invasiveness of current therapeutic approaches. Future strategies should focus on personalized treatments that address multiple mutations, which are critical to improving patient outcomes [188].

Studies have shown that METTL14 expression is downregulated in RP patients, and its silencing leads to a significant reduction in m6A levels in RPE cells. This reduction, in turn, impairs cellular phagocytosis and proliferation, while promoting apoptosis and cell cycle arrest. RNA-seq and MeRIP-seq analyses have revealed that METTL14 regulates the expression of microtubule-associated protein 2 (MAP2) through m6A modification. Notably, MAP2 overexpression replicates the effects of METTL14 silencing, resulting in RPE cell dysfunction and upregulation of NEUROD1 expression, a pathogenic gene associated with RP [189].

Mutations in the RP switch gene lead to rod death, followed by a gradual loss of cones. Cones are essential for color vision and sharp visual acuity; their degeneration results in impaired color vision and blurred sight [187]. The involvement of the mTOR pathway in cone survival in RP is well-documented [190]. Moreover, m6A methylation has been closely associated with the activity of the mTOR pathway [191].

These studies underscore the pivotal role of m6A methylation in the pathogenesis of RP, supporting the development of novel therapeutic strategies that target m6A regulation. Future research is essential to further explore the specific relationship between m6A modification and retinal cell function, as well as how modulation of m6A levels can protect cones and slow disease progression.

Since RP is a genetic disorder primarily caused by mutations, fetuses carrying causative mutations could theoretically be identified through genetic testing during pregnancy, such as Non-Invasive Prenatal Testing (NIPT) or amniocentesis. However, RP involves over 70 genes and more than 3000 mutation types, and its genetic heterogeneity means that routine testing fails to identify the causative locus in approximately 40% of patients [188]. Even when mutations are detected, current clinical interventions are limited to delaying disease progression (e.g., vitamin A supplementation, retinal prostheses), rather than offering a cure [192]. RP typically begins gradually after birth, and effective fetal interventions remain unavailable. Future research is crucial to identify early diagnostic markers for RP and to develop safe and effective fetal intervention strategies, enabling the detection of high-risk fetuses and improving patient outcomes.

#### Proliferative vitreoretinopathy (PVR)

PVR is a major complication following retinal detachment (RD) surgery, occurring in approximately 5%–10% of cases. It is characterized by the formation of contractile fibrous membranes on the vitreous and retinal surfaces, often leading to surgical failure and redetachment. The pathogenesis of PVR is complex, primarily involving the migration and proliferation of RPE and glial cells, along with excessive deposition and contraction of the extracellular matrix (ECM) [193]. The inflammatory response also plays a critical role in promoting cell proliferation and fibrous membrane formation through the release of various growth factors and mediators. Currently, treatment for PVR largely relies on the surgical removal of fibrous membranes to relieve traction, but the success rate remains limited, and the recurrence rate is high. Although some drugs have demonstrated efficacy in animal models, clinical trials have largely been unsuccessful, and effective preventive and therapeutic options are still lacking [194]. Therefore, a deeper understanding of the pathogenesis of PVR and the identification of new therapeutic targets are crucial for improving patient outcomes.

Epithelial-mesenchymal transition (EMT) of RPE cells plays a critical role in the development of PVR [195]. Recent studies have highlighted the regulatory role of m6A modification in EMT in RPE cells. TGF-β2 has been shown to influence the EMT process in RPE cells by regulating m6A modification, with METTL3 and YTHDF1 playing key roles in cell migration and the expression of EMT markers [196]. Furthermore, MeCP2 inhibits EMT in RPE cells by regulating m6A modification and the expression of key genes, such as EGR1 [197]. Overexpression of METTL3 has been found to significantly delay PVR progression by inhibiting the Wnt/β-catenin signaling pathway [198]. These findings suggest that EMT in RPE cells during PVR may be complex, influenced by multiple signaling pathways and epigenetic regulators. Further studies are needed to explore these mechanisms in greater detail.

#### Age-related macular degeneration (AMD)

AMD is the leading cause of blindness in individuals over 60 in industrialized countries, primarily affecting the macular region of the retina. This area, rich in cones, is responsible for central vision and plays a crucial role in tasks requiring fine visual acuity, such as reading, driving, and recognizing faces [199]. In 2020, more than 190 million people worldwide were affected, and with the global aging trend, this number is expected to rise to 288 million by 2040. The disease not only severely impacts vision but also places a significant burden on global healthcare systems [200].

The pathogenesis of AMD is complex, involving multiple factors, including genetic susceptibility, environmental influences such as smoking, diet, and lifestyle, as well as age-related physiological changes. Chronic inflammation, lipid deposition, oxidative stress, and impaired ECM maintenance play pivotal roles in the development of AMD. The disease affects photoreceptors, retinal RPE, Bruch’s membrane (BrM), and the choriocapillaris complex. Pathological features of AMD include the formation of lipoprotein deposits (drusen) beneath the RPE and degenerative changes in both the RPE and choriocapillaris layer [201]. Advanced AMD is classified into atrophic AMD (dry AMD), characterized by RPE and neurosensory retinal atrophy, and neovascular AMD (wet AMD), characterized by choroidal neovascularization, which may lead to subretinal or intraretinal fluid accumulation and hemorrhage. Although wet AMD accounts for only 10%–15% of cases, it remains the leading cause of severe vision loss.

No effective treatment has been developed for dry AMD, and its exact mechanism remains unclear [202]. Targeting the complement pathway, particularly by inhibiting Complement Factor D (CFD), has shown significant promise in slowing the progression of dry AMD [203]. On the other hand, although intravitreal anti-VEGF injections are the standard treatment for wet AMD, frequent injections can result in adverse reactions such as glaucoma, uveitis, and cataracts, as well as poor patient compliance. As a result, researchers are actively exploring alternative treatment strategies [84]. In the field of epigenetics, m6A modification has emerged as a key area of interest. Associations between m6A modifications and specific pathological features in AMD have been identified, offering potential novel therapeutic targets.

RPE is the primary site of AMD pathology, and its dysfunction is closely associated with AMD progression [204]. Chen et al. found that circRNA circSPECC1 was significantly downregulated in RPE cells from AMD patients, leading to oxidative stress, ferroptosis, mitochondrial dysfunction, lipid metabolism disorders, and impaired phagocytic function. In mouse models, the loss of circSPECC1 further resulted in decreased visual acuity, RPE abnormalities, and disruption of retinal homeostasis. Mechanistic studies revealed that m6A modification of circSPECC1 regulates its nuclear export through YTHDC1. CircSPECC1 acts as a sponge for miR-145-5p, preventing its interaction with Cyclin-dependent kinase inhibitor 1A (CDKN1A) and regulating RPE cell function. Notably, overexpression of miR-145-5p exacerbates oxidative damage and dysfunction in RPE cells, whereas its inhibition reverses the negative effects caused by circSPECC1 deletion [202].

Aβ induces degeneration of the RPE and retina. Li et al. demonstrated the protective role of FTO in regulating Aβ1-40-induced RPE degeneration. Their study showed that FTO expression was significantly upregulated in Aβ1-40-treated RPE cells. Inhibition of FTO was found to activate the PKA/cAMP signaling pathway, thereby exacerbating RPE cell degeneration [205].

Aberrant activation of the Wnt signaling pathway has been observed in the macular tissue of AMD patients [206]. Additionally, METTL3 regulates pathological angiogenesis in the retina through aberrant activation of the Wnt signaling pathway, highlighting its critical role in the pathogenesis of AMD [80]. Meanwhile, microtubule-associated protein 2 (MAP2) is highly expressed in the retinas of AMD patients [207]. Further studies demonstrated that silencing METTL14 promotes the translation of MAP2 mRNA, leading to reduced phagocytosis, disruption of tight junctions, increased apoptosis, and cell cycle arrest. Additionally, YTHDF2, an m6A reading protein, weakens its binding to and the degradation of MAP2 mRNA due to the reduced m6A modification caused by METTL14 silencing, resulting in increased MAP2 expression. YTHDF2 exhibited similar effects to METTL14 in the development of AMD. This effect may influence the pathological process of AMD by regulating the stability and translational efficiency of MAP2 mRNA [189].

Currently, there are no curative treatments for AMD, and existing therapies primarily focus on delaying disease progression and preserving vision. As such, preventive measures are crucial, including smoking cessation, maintaining a balanced diet, weight control, reducing UV exposure, and regular fundus examinations for individuals over 50. Although studies have emphasized the significant role of m6A modification in AMD, its specificity, interaction with other epigenetic modifications, and potential as a therapeutic target require further investigation. Additionally, AMD affects the RPE, BrM, and choriocapillaris complex. While the RPE has received substantial attention, research into the BrM and choriocapillaris complex is equally critical. Future treatments may not only involve RPE transplantation but also the repair or replacement of Bruch’s membrane and the choriocapillaris complex [3].

#### Traumatic optic neuropathy (TON)

TON is an optic nerve injury resulting from head or eye trauma, often leading to severe visual impairment or blindness. It can be classified into direct injury (e.g., damage to the optic nerve anatomy) and indirect injury (e.g., compression or traction from closed trauma). Due to its unique anatomical location, the optic canal and intracranial segments are particularly vulnerable to damage from external forces. The pathophysiological mechanism involves both primary injury (axonal tear) and secondary injury (edema, ischemia, and apoptosis in the optic canal) [208]. Clinical manifestations include decreased visual acuity, relative afferent pupillary defects, visual field defects, and changes in the optic disc [209].

Studies by Qu et al. were the first to reveal dynamic changes in m6A modification in the rat retina following TON. The expression of m6A-related genes, including METTL3, WTAP, FTO, and ALKBH5, was significantly upregulated in the retina 12 h after optic nerve clamping in rats, suggesting that m6A modification plays a critical role in the early pathophysiology of TON. MeRIP-seq analysis identified 2810 upregulated m6A peaks and 689 downregulated peaks following TON, with the majority concentrated in CDS regions, indicating that m6A regulates gene expression by influencing mRNA stability and translational efficiency. GO analysis showed that upregulated m6A peaks were closely associated with nervous system development, protein binding, and intracellular components, while downregulated m6A peaks were linked to cellular localization and the unfolded protein response. KEGG pathway analysis further revealed that upregulated m6A peaks were significantly enriched in MAPK, NF-κB, and TNF signaling pathways, which are crucial in neuroinflammation and nerve injury [210].

While clipping the optic nerve effectively mimics TON caused by ocular trauma, it can also result from head trauma. Akhter et al. developed a TON model of optic nerve injury caused by head trauma (e.g., impact) by fixing mice with a stereotaxic instrument, exposing the optic nerve via medial canthotomy, and simulating head impact with an impactor [211]. Ryan et al. introduced a new animal model of TON, Torsion-Induced Traumatic Optic Neuropathy (TITON), which induced significant visual dysfunction by rapidly rotating the rat eye to simulate the torsional shear forces on the optic nerve during closed head trauma [212]. Future studies should thoroughly investigate changes in m6A modification levels and their regulatory mechanisms in these models to clarify the pathological mechanisms of TON and provide a theoretical foundation for targeted therapies.

Recent studies have shown that WTAP is significantly upregulated in neurons during the early stages of traumatic brain injury (TBI). This upregulation promotes the activation of the NLRP3 inflammasome by directly targeting the m6A modification site on NLRP3 mRNA, thereby exacerbating neuroinflammation and neurological dysfunction. Furthermore, as a “reader” of m6A, YTHDF1 can recognize the m6A modification on NLRP3 mRNA and enhance NLRP3 protein expression through translational regulation. Downregulation of either WTAP or YTHDF1 significantly reduced nerve injury and inflammatory responses following TBI [213].

Recent studies have highlighted the critical role of the m6A demethylase ALKBH5 in axonal regeneration, as demonstrated in rat sciatic nerve compression and mouse optic nerve compression models. In the peripheral nervous system (PNS), ALKBH5 regulates the stability of Lpin2 mRNA through m6A modification. As a key regulator of lipid metabolism, downregulation of Lpin2 expression helps neurons acquire sufficient lipids during regeneration, thereby promoting axonal regeneration. In the central nervous system (CNS), knockdown of ALKBH5 similarly enhanced retinal ganglion cell (RGC) survival and axonal regeneration, suggesting that ALKBH5 exerts inhibitory effects in both the CNS and PNS. Moreover, selective inhibitors of ALKBH5 have been shown to promote axonal regeneration in neurons [214].

These findings provide a crucial rationale for functional recovery following nerve injury. However, most of these studies are based on animal models, which differ significantly from the human nervous system, posing challenges for clinical translation.

### Ocular tumors

Ocular tumors are serious diseases that threaten both sight and life, primarily affecting the eyelid, conjunctiva, uveal tract, retina, and orbit. Common types include basal cell carcinoma of the eyelid, conjunctival squamous cell carcinoma, uveal melanoma, and retinoblastoma, which are associated with factors such as UV exposure and genetic mutations. In recent years, advancements in molecular mechanism research have led to the development of new therapeutic strategies, offering renewed hope for patients [215, 216].

#### Ocular melanoma

Ocular melanoma is a malignant tumor originating from intraocular melanocytes, including conjunctival melanoma (CM) and uveal melanoma (UM). It is the second most common type of melanoma, following cutaneous melanoma [217]. The incidence is approximately 1–2 cases per million people annually worldwide. Unlike cutaneous melanoma, the development of ocular melanoma is minimally associated with UV exposure. The genetic mutations characteristic of ocular melanoma are primarily GNAQ and GNA11 mutations, which activate intracellular signaling pathways and promote tumorigenesis. Ocular melanoma has a short median survival, particularly in metastatic patients. Early diagnosis and treatment are critical for improving prognosis [218, 219].

##### Conjunctival melanoma (CM)

Jia et al. found that global m6A levels were decreased in ocular melanoma, which correlated with reduced translation efficiency of the tumor suppressor gene histidine triad nucleotide-binding protein 2 (HINT2) mRNA. This suggests that m6A modification plays a crucial role in the suppression of ocular melanoma [220]. Furthermore, He et al. revealed the cancer-promoting mechanism of m6A modification in ocular melanoma, showing that increased m6A modification of beta-secretase 2 (BACE2) mRNA enhanced its translational efficiency. BACE2 regulated ER calcium release by influencing the expression of transmembrane protein 38B (TMEM38B), which in turn promoted the proliferation and migration of tumor cells [221].

In recent years, the critical role of the tumor microenvironment (TME) in CM progression has garnered increasing attention. Among these, cancer-associated fibroblasts (CAFs), a key component of the TME, significantly influence tumor angiogenesis and malignant progression through complex cell-cell interactions and extracellular matrix remodeling. Recently, Liao et al. demonstrated that CAFs in the CM microenvironment upregulate FTO expression, which stabilizes and enhances VEGFA and EGR1 mRNA expression by removing m6A modifications, thereby promoting tumor angiogenesis. Furthermore, high FTO expression is strongly associated with poor prognosis in CM patients, suggesting its potential as both a prognostic marker and therapeutic target [222].

High expression of RNA-binding motif single-stranded interacting protein 1 (RBMS1) is associated with increased immune cell infiltration and elevated expression of Programmed Cell Death-Ligand 1 (PD-L1) in the TME, suggesting its role in immunosuppression. Additionally, RBMS1 promotes tumor progression by regulating cell proliferation, apoptosis, and ferroptosis in tumor cells. Its silencing significantly inhibits ocular melanoma proliferation and migration while promoting apoptosis [223]. Encouragingly, RBMS1 also enhances S100 calcium-binding protein P (S100P) mRNA translation and accelerates metastasis in non-small cell lung cancer (NSCLC) cells by interacting with YTHDF1, which is associated with poor prognosis in NSCLC [224].

Furthermore, enhancer of zeste homolog 2 (EZH2) is significantly overexpressed in CM, particularly in primary tumors and lymph node metastases. Its expression level is closely linked to tumor thickness and poor prognosis. In vitro experiments have shown that inhibiting EZH2 through drugs or gene knockdown significantly reduces CM cell proliferation and colony formation, while inducing cell cycle arrest and apoptosis [225]. Interestingly, m6A modification-mediated upregulation of EZH2 has been found to be associated with various pathological processes, such as Primary Sjögren’s Syndrome and osteolytic bone metastasis in breast cancer [226, 227].

Future studies should further explore the interaction between m6A modification, RBMS1, and EZH2, with a particular focus on their potential link in ocular melanoma.

##### Uveal melanoma (UM)

Uveal melanoma (UM) is the most common type of ocular melanoma, accounting for about 85% of cases. Half of the patients will eventually develop metastases, with the liver being the primary site of metastasis [228]. Patients with metastases have a very low 1-year survival rate, with only 15% surviving [229]. Therefore, there is an urgent need to identify effective biomarkers and therapeutic targets for UM.

In addition to YTHDF1 and METTL3, which affect ocular melanoma by influencing their downstream target genes, FTO has also been found to play a key role in ocular melanoma [220, 230]. FTO expression was significantly upregulated in UM. In vitro experiments showed that FTO knockdown significantly increased proliferation, migration, and invasion of UM cells, while inhibiting apoptosis. This suggests that FTO may act as a tumor suppressor in UM. Further studies revealed that FTO inhibited autophagy-related gene 5 (ATG5) expression by directly targeting its m6A modification site, affecting autophagosome formation and function. Moreover, ATG5 overexpression significantly reversed the promoting effect of FTO knockdown on UM cell proliferation and metastasis, further confirming the critical role of ATG5 in FTO regulation of UM progression [231].

Tian et al. developed a novel FTO-targeted nanomedicine, SNAMA (Self-loaded Nucleic Acid MA-assembled nanoparticle), to target the key role of FTO in UM. The FTO inhibitor meclofenamic acid (MA) regulates transcriptional processes dependent on m6A modification and induces a unique form of cell death, disulfidptosis, in UM cells. However, MA is limited in clinical applications due to its low bioavailability and poor tumor targeting. To address these limitations, the investigators designed MA-loaded nucleic acid nanomedicine SNAMA, which releases MA through glutathione (GSH) and activates m6A-mediated disulfidptosis, significantly inhibiting UM cell growth. Further studies revealed that SNAMA-apt, formed by binding the PD-L1 aptamer to the outer layer of SNAMA, significantly enhanced its targeting ability and immunomodulatory effect in UM. In orthotopic and liver metastasis models of UM, both SNAMA and SNAMA-apt exhibited significant antitumor effects, effectively reducing tumor burden and prolonging survival [232].

METTL14, ALKBH5, and METTL3 are highly expressed in UM and are strongly associated with poor patient prognosis. METTL14 regulates the stability of Runt-Related Transcription Factor 2 (RUNX2) mRNA through m6A modification, activating the Wnt/β-catenin signaling pathway and promoting UM cell migration and invasion [233]. ALKBH5 drives EMT in UM cells by demethylating Forkhead box protein M1 (FOXM1) mRNA, increasing its expression and stability, thereby promoting tumor progression. In vitro and in vivo experiments showed that ALKBH5 knockdown significantly inhibited UM cell proliferation, migration, and invasion, while promoting apoptosis [234]. METTL3, the primary m6A regulatory enzyme, promotes UM cell proliferation and invasion by regulating the translation of c-Mesenchymal-Epithelial Transition Factor (c-Met) protein. Inhibition of m6A modification significantly reduces phosphorylated Akt (p-Akt) levels, cell cycle-related proteins, and UM cell function [235].

In addition, the regulatory mechanisms of circRNAs have garnered increasing attention. circ0053943 enhances EGFR mRNA stability regulation by IGF2BP3 through interaction with the KH1 and KH2 domains of the IGF2BP3 protein. This process depends on m6A modification; IGF2BP3 stabilizes the transcript by recognizing the m6A modification site on EGFR mRNA, thereby upregulating EGFR protein expression. This m6A modification-dependent mechanism activates the MAPK/ERK signaling pathway, significantly promoting the proliferation, migration, and invasion of UM cells [236].

By integrating pan-cancer data from TCGA, TARGET, and GTEx, the expression patterns of Atypical Chemokine Receptor 2 (ACKR2) across 34 cancers and its correlation with Overall Survival (OS) and Progression-Free Interval (PFI) were analyzed. High ACKR2 expression in UM was significantly associated with a favorable prognosis, and its expression was regulated by gene copy number variation, BAP1 gene mutation frequency, and DNA and RNA modifications [237]. Furthermore, based on UM patient data from The Cancer Genome Atlas (TCGA), RBM15B was identified as the only independent prognostic factor in UM. Its high expression was significantly associated with better OS, Disease-Specific Survival (DSS), and PFI, and negatively correlated with multiple immune checkpoints [238]. Further analysis revealed that m6A regulators and their associated lncRNAs play a crucial role in tumor microenvironment remodeling in UM. Risk signatures based on RBM15B, YTHDF3, and IGF2BP2 effectively distinguish low-risk groups with good prognosis from high-risk groups with poor prognosis [239].

A comprehensive analysis of UM samples from the TCGA and GEO databases revealed that UM can be classified into two distinct molecular subtypes, C1 and C2, based on the expression of m6A regulators. The C2 subtype was associated with a more favorable prognosis, characterized by a higher proportion of type 1 subtype and a lower frequency of monosomy 3. Moreover, a prognostic signature comprising three m6A regulators—ALKBH5, YTHDF1, and KIAA1429—was developed through multivariate Cox regression analysis. This signature robustly predicted overall survival and demonstrated a strong correlation with both chromosome 3 status and the type 1 subtype of UM [240].

These findings suggest that the expression levels of ACKR2 and RBM15B may serve as potential prognostic markers for UVM and LIHC. Furthermore, m6A regulators and their associated lncRNAs could influence UM prognosis by modulating immune responses and the tumor microenvironment. However, experimental validation is required to substantiate these results. Future research should aim to confirm these observations through in vitro cell experiments and in vivo animal models, thereby providing a solid scientific foundation for the diagnosis and treatment of UM.

#### Retinoblastoma (RB)

Retinoblastoma (RB) is an aggressive pediatric malignancy originating from immature retinal cells, caused by bi-allelic inactivation of the Retinoblastoma 1 (RB1) gene. It is the most common intraocular malignancy in children, with approximately 9000 new cases diagnosed annually worldwide, and an incidence rate of 1 in 15,000–20,000 live births. Although the disease has a high cure rate in developed countries, mortality remains as high as 70% in low- and middle-income countries, primarily due to limited public awareness and insufficient healthcare resources [241, 242]. The most common early sign of RB is leucocoria, which presents as white reflections in the pupillary area. Other symptoms include strabismus, decreased visual acuity, and glaucoma. Treatment approaches involve local therapies such as laser and cryotherapy, systemic chemotherapy, radiation therapy, and enucleation when necessary [243]. Recent advancements in genetic testing technologies have significantly enhanced our understanding of the genetic and molecular mechanisms underlying RB, opening new avenues for precision treatment strategies.

Recent studies have demonstrated that METTL14 enhances the stability of LINC00340, a long non-coding RNA, through m6A modification. This process activates the Notch signaling pathway, thereby promoting RB cell proliferation and inhibiting apoptosis [244]. Furthermore, the deubiquitinating enzyme Ubiquitin-specific peptidase 49 (USP49) is highly expressed in RB, where it enhances autophagy by stabilizing Sirtuin 1 (SIRT1) protein. This stabilization promotes RB cell proliferation and contributes to resistance to chemotherapeutic agents, such as carboplatin (CBP). Subsequent investigations have revealed that IGF2BP3 upregulates USP49 expression through m6A modification, thereby enhancing SIRT1 stability and underscoring the critical role of m6A modification in RB resistance [245].

A recent study revealed that FTO is highly expressed in RB tissues, as demonstrated by bioinformatics analysis, and is negatively correlated with m6A levels. Further in vitro experiments showed that FTO knockdown significantly inhibited RB cell proliferation, migration, and invasion, while causing cell cycle arrest at the G0/G1 phase. Mechanistic investigations indicated that FTO influences RB progression by regulating m6A modification and stabilizing E2F3. E2F3, a crucial regulator of the cell cycle, exhibited a marked increase in m6A modification levels following FTO knockdown, which led to reduced mRNA stability. Additionally, animal studies confirmed the inhibitory effect of FTO knockdown on RB tumor growth. This effect was partially reversed by overexpressing E2F3, further supporting the molecular mechanism by which FTO modulates RB progression through E2F3 [246].

Chen and Zeng’s team utilized the GSE97508 dataset to identify genes with significant differential expression in RB samples. Their analysis revealed notable enrichment of genes associated with the p53 signaling pathway, including CDKN2A, CCNB2, and RRM2, as determined through KEGG enrichment analysis. Of particular interest, the expression of Cyclin-dependent kinase inhibitor 2A (CDKN2A) was found to be especially prominent. Building on this bioinformatics analysis, the team further validated their findings through in vitro experiments. They discovered that silencing CDKN2A markedly inhibited RB cell proliferation and induced apoptosis, an effect closely linked to the activation of the p53 pathway. Additional studies demonstrated that METTL14 stabilizes CDKN2A expression through m6A modification, thereby promoting the malignant behavior of RB cells [247].

Recent research has unveiled a novel mechanism through which the N-myc Proto-Oncogene Protein (MYCN) promotes RB cell proliferation and tumor growth. This occurs via the upregulation of YTHDF1. MYCN directly interacts with the promoter region of YTHDF1, leading to a significant increase in its expression in RB cells and subsequent transcriptional activation. The overexpression of YTHDF1 enhances both RB cell proliferation and tumor growth by stabilizing and facilitating the translation of key oncogene mRNAs, including CDK5R1, RBM15, MAT2A, and CNST. Notably, the m6A-binding ability of YTHDF1 is essential for its oncogenic function. A mutant variant of YTHDF1, in which Lysine 395 is replaced by Alanine (K395A), was unable to restore the proliferative capacity in YTHDF1 knockdown cells. This finding underscores the critical role of m6A modification in mediating the oncogenic activity of YTHDF1 [248].

Additionally, METTL3 is significantly overexpressed in RB tissues and cell lines, where it promotes RB cell proliferation, migration, invasion, and colony formation through the regulation of key components within the PI3K/AKT/mTOR signaling pathway. These components include the phosphorylation of PI3K-p85, AKT, mTOR, P70S6K, and 4EBP1. Animal model experiments further support the notion that METTL3 expression is closely linked to the growth of RB tumors [191].

**Fig. 4:**
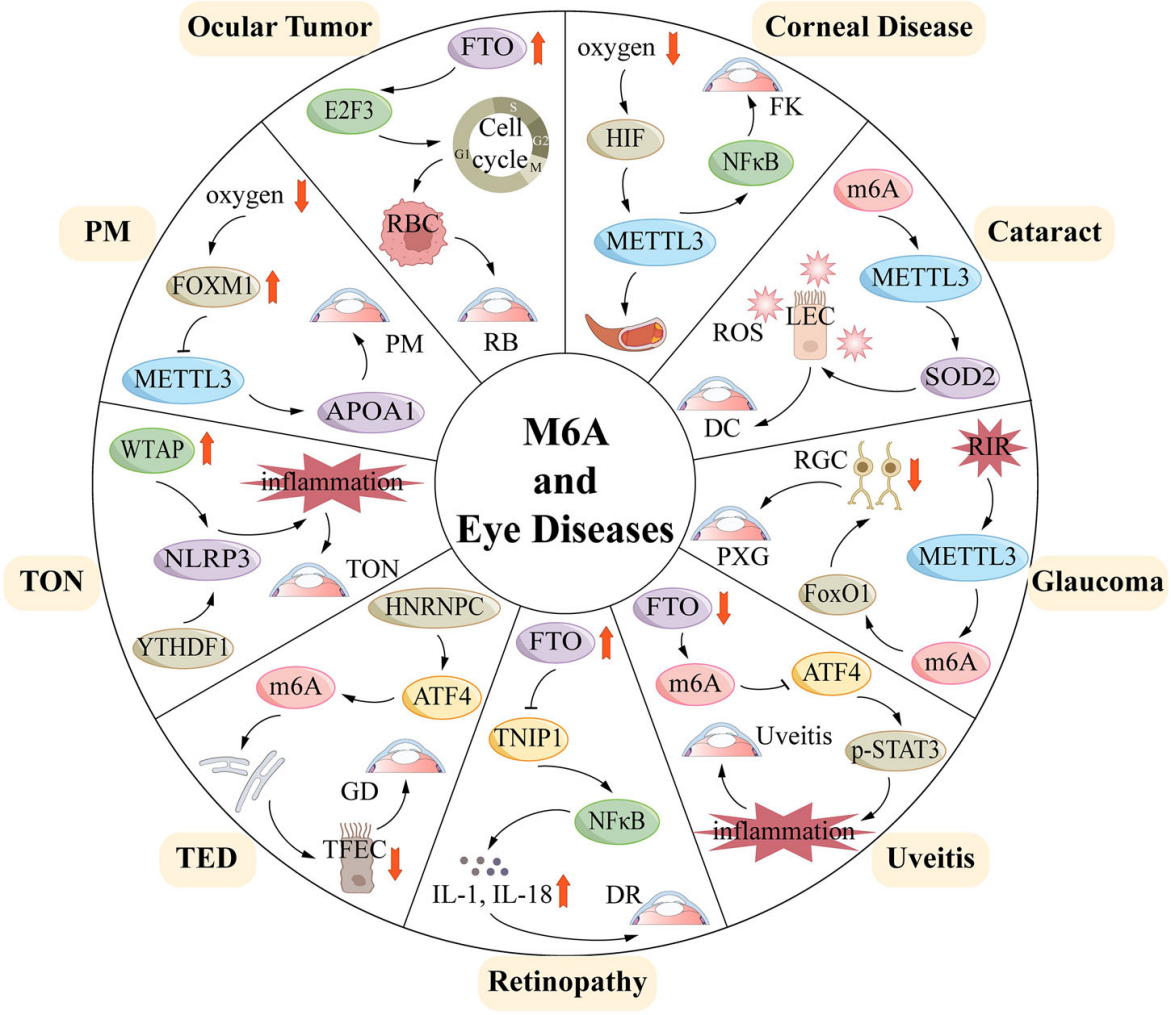
Specific mechanisms by which m6A is associated with eye diseases. In ocular tumors, such as retinoblastoma (RB), m6A regulates the cell cycle progression of retinoblastoma cells (RBC) and lens epithelial cells (LEC) by modulating the E2F3/RB pathway and hypoxia-inducible factor (HIF) [246]. In diabetic eye diseases, FTO regulates FoxO1 and NF-κB in an m6A-dependent manner, driving the pathological processes of diabetic retinopathy (DR) and diabetic cataract (DC) [3]. In inflammatory and immune eye diseases, m6A modification regulates the NLRP3 inflammasome, NF-κB pathway, and TNIP1, contributing to the pathogenesis of uveitis, fungal keratitis (FK), and thyroid-associated ophthalmopathy (TED)/Graves’ ophthalmopathy (GD) [141]. In glaucoma and optic nerve injury, m6A affects oxidative stress and FoxO1 signaling, participating in retinal ganglion cell (RGC) damage in primary open-angle glaucoma (PXG), ocular hypertension (TON), and traumatic optic neuropathy (TON) [2]. Additionally, m6A plays a key role in pathologic myopia (PM) through the HIF pathway in a hypoxic environment and in cataract formation by regulating ATF4 endoplasmic reticulum stress and SOD2 antioxidant defense mechanisms [299]. DC diabetic cataract, DR diabetic retinopathy, GD Graves’ disease, FK fungal keratitis, FTO fat mass and obesity associated protein, HIF hypoxia-Inducible factor, LEC lens epithelium cell, PM pathologic myopia, RB retinoblastoma, RBC retinoblastoma cell, RGC retinal ganglion cell, RIR retinal ischemia-reperfusion, ROS reactive oxygen species, TED thyroid eye disease, TON traumatic optic neuropathy.

Future studies should focus on elucidating the dynamic regulatory mechanisms underlying m6A modification, thereby establishing a foundation for the development of more targeted treatment strategies. Additionally, investigating the clinical applications of m6A modification in RB, particularly its potential role as an early diagnostic marker or therapeutic target, will be a crucial direction for future research (Fig. 4).

### Disorders with pan-ocular involvement: pathologic myopia (PM)

Myopia is one of the most prevalent eye problems worldwide, and as early as 2015, Dolgin predicted an explosive increase in its global prevalence. By 2050, it is estimated that nearly half of the world’s population will have myopia, with around 10% potentially developing high myopia [249]. High myopia is defined as a spherical equivalent (SE) worse than −5.0 or −6.0 diopters (D), or an axial length ≥26 mm. Pathological myopia (PM) refers to high myopia accompanied by pathological retinal changes, which can lead to severe ocular complications such as retinal detachment, macular degeneration, and cataracts. These complications can significantly threaten patients’ visual health and may even result in blindness [10, 250].

Apolipoprotein A1 (APOA1), a major component of high-density lipoprotein, is involved in reverse cholesterol transport and plays a crucial role in inhibiting axial overgrowth. It is also considered a potential target for PM therapy [251, 252]. Xue et al. developed a myopic cell model through hypoxic treatment and found that APOA1 and Forkhead box M1 (FOXM1) expression were upregulated, while METTL3 expression was downregulated in Human Scleral Fibroblasts (HSFs) under hypoxic conditions. Further studies revealed that FOXM1 inhibited its own transcription by binding to the METTL3 promoter, thereby increasing the m6A modification level of APOA1 and enhancing APOA1 mRNA stability and transcription. YTHDF2, an m6A “reader,” influences APOA1 expression by recognizing m6A-modified APOA1 mRNA and regulating its degradation rate. Moreover, FOXM1 knockdown reversed the inhibition of HSF proliferation and the promotion of apoptosis caused by hypoxic treatment. In summary, FOXM1 enhances and stabilizes APOA1 expression by inhibiting METTL3 and YTHDF2, which in turn affects scleral remodeling [253]. The sclera, as the primary load-bearing connective tissue of the eye, undergoes remodeling that plays a crucial role in the development and progression of myopia [254, 255].

Notably, APOA1 is recognized as a “STOP” signal that slows myopia progression by inhibiting axial overgrowth and may also play a role in the transport of retinoic acid (RA) from the choroid to the sclera, which inhibits scleral proteoglycan synthesis and prevents myopia development [251, 256]. In pathological myopia, the upregulation of APOA1 expression may be limited by various factors. On one hand, its upregulation may serve as a protective response to increased intraocular oxidative stress, but it is insufficient to fully prevent myopia progression. On the other hand, its function may be regulated by factors such as m6A modification, which weakens its protective effect. Future studies should further investigate the role of APOA1 in pathological myopia and establish a foundation for developing new treatment strategies.

The study by Wen et al. is the first to map the m6A modification of the anterior lens capsule in patients with high myopia, revealing its potential role in the pathomechanism. The m6A modification site was significantly increased in the anterior lens capsule of patients with high myopia compared to those with simple nuclear cataract. These modifications were primarily concentrated in the stop codon, coding sequence (CDS), and 3’ untranslated region (3’ UTR) of mRNA. The modified genes were enriched in pathways related to ECM formation, suggesting that m6A modification may influence fundus structure by regulating ECM components. Additionally, the expression of m6A-related enzymes, particularly the demethylases ALKBH5 and FTO, was downregulated, while methyltransferase METTL14 was upregulated in patients with high myopia. This imbalance may result in hypermethylation of chitinase-like protein 1 (CHI3L1), affecting YKL-40 protein expression and regulating ECM composition, thereby promoting the pathological state of high myopia [10].

Notably, the study by Li et al. found that ALKBH5 was significantly upregulated in ARC patients [111]. Similarly, Lim and Chandra et al. identified a strong association between FTO and an increased risk of cataracts using different research methods [109, 110]. However, why is ALKBH5 and FTO expression downregulated in nuclear cataract patients with high myopia? Does high myopia lead to the downregulation of ALKBH5 and FTO expression by activating other signaling pathways? Answers to these questions are crucial for gaining a deeper understanding of the molecular mechanisms linking high myopia and cataracts and warrant further investigation.

tRNA-derived fragments (tRFs) are a novel class of small non-coding RNAs that play crucial roles in various biological and pathological processes [257, 258]. Liu et al. investigated the role of tRF-228BWS72092 (tRF-22) in choroidal vasculopathy and PM progression. tRF-22 expression was downregulated in the choroid of myopic eyes, while its overexpression significantly inhibited myopia progression, improving refraction and slowing axial elongation. Mechanistic studies revealed that tRF-22 enhances protein expression by targeting METTL3 mRNA and inhibiting its expression, which in turn reduces m6A modification of Axin1 and Arid1b mRNA. Axin1 and Arid1b function as negative regulators of the Wnt signaling pathway, and their increased expression suppresses pathway activation, thereby alleviating choroidal vasculopathy [259].

## Treatment and future prospects

Due to the small size of the eye and its relatively autonomous internal environment, therapeutic agents can be precisely delivered to targeted areas through suprachoroidal, intravitreal, or subretinal injections. This method ensures that effective concentrations are maintained over time, minimizing the required quantities of reagents.

The eye, being a non-regenerative organ, presents both ethical and technical challenges in obtaining tissue samples for single-cell analysis [260]. Recently, the development of liquid biopsy techniques has provided promising solutions to these issues [261]. Liquid biopsy offers a non invasive or minimally invasive means of reflecting tissue pathological conditions by analyzing biomarkers present in body fluids [262]. However, traditional liquid biopsy methods are limited by their resolution at the cellular level, which hampers the precise identification of the cellular origins of disease-associated proteins [263].

To address this limitation, Wolf et al. introduced an innovative multi-omics approach called TEMPO (Tracing Expression of Multiple Protein Origins), which integrates liquid biopsy proteomics, single-cell transcriptomics, and artificial intelligence techniques. TEMPO successfully traced the cellular origins of over 5900 proteins by analyzing proteins in small volumes of body fluids, alongside single-cell transcriptomic data. This integration allows for precise in vivo assessment of disease mechanisms at the cellular level. The technique has provided valuable insights into disease-stage-specific cellular activities in ophthalmic conditions such as RP and DR, and has identified disease-associated neural cell proteins in the aqueous humor of Parkinson’s disease (PD) patients. These findings underscore the potential of the eye as a “window” for evaluating the status of brain diseases [260].

In addition, the TEMPO method provides a novel perspective on studying organ aging. The proteome of the eye exhibits a complex, nonlinear trajectory of changes during normal aging, primarily associated with specific cell types. Using artificial intelligence models, the researchers predicted the “Molecular Age” of the eye and discovered that specific cell types in patients with non-age-related diseases, such as DR and RP, exhibited signs of accelerated aging. This finding suggests a potential link between aging and disease, opening new avenues for developing therapeutic strategies targeting aging-related mechanisms.

Mutations in the Crumbs Homolog 1 (CRB1) gene are strongly associated with a range of inherited retinal degenerative diseases, including Leber Congenital Amaurosis (LCA) and RP. These conditions are characterized by the progressive degeneration of photoreceptor cells and subsequent vision loss, with their pathogenesis being a focal point of research. Recent studies have provided new insights, revealing that CRB1 plays a crucial role in the retina and is also involved in maintaining the barrier integrity of the colonic epithelium. Mutations in the CRB1 gene disrupt epithelial barrier function in both the retina and the colon, facilitating the translocation of intestinal bacteria to the retina via the bloodstream, which triggers inflammation and retinal degeneration [264]. These findings offer a rationale for developing antibiotic-based treatment strategies, providing new hope for the treatment of these blinding diseases.

**Fig. 5:**
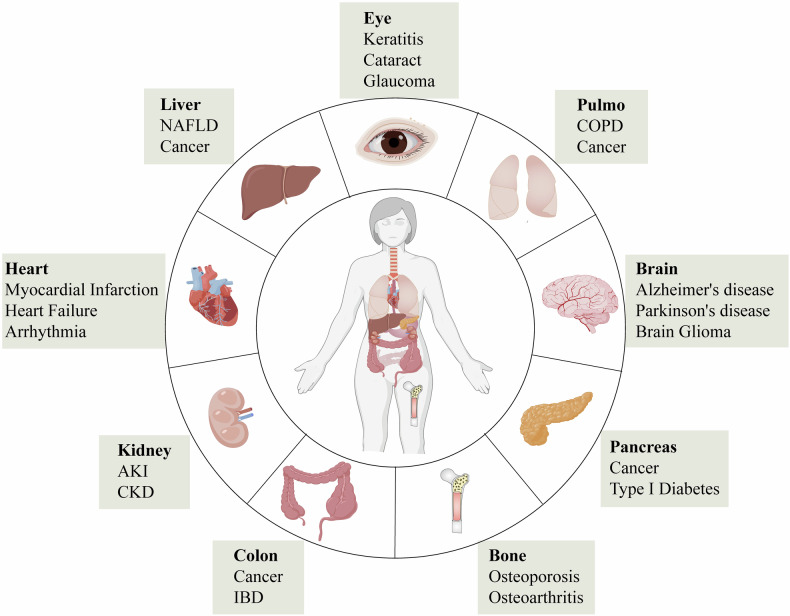
M6A is associated with various tissue and organ diseases. AKI acute kidney disease, CKD chronic kidney disease, COPD chronic obstructive pulmonary disease, IBD inflammatory bowel disease, NAFLD non-alcoholic fatty liver disease.

As the most abundant RNA modification, m6A plays a critical role in various pathological processes (Fig. 5). We summarize its involvement in several ocular diseases (Table 3). Due to its significant role, m6A has emerged as a potential therapeutic target, with ongoing research focused on developing drugs to regulate cellular function.

**Table 3:** The roles of m6A modifications in complex ocular diseases.

Diseases	Test object	m6A	Proteins	Pathway	Roles in eye diseases	Ref.
Keratitis	1. BALB/c mice 2. Mouse Corneal Stromal Cells	——	METTL3↑	METTL3-PI3K/Akt	Exacerbates inflammatory response, worsening keratitis.	[99]
	1. BALB/c mice 2. Mouse Corneal Tissue	↑	METTL3↑	METTL3-PI3K/Akt	Promotes the occurrence and progression of keratitis.	[100]
	1. BALB/c mice 2. Mouse corneal stromal cells	↑	METTL3↑	METTL3-TRAF6-p-IκB/IκB-p-p65/p65-NF-κB	Exacerbates the inflammatory response, worsening keratitis.	[101]
Corneal neovascularization	1. HUVECs 2. OIR model, mouse corneal alkali burn model 3. METTL3 knockout mice	↑	METTL3↑	METTL3-m6A-LRP6/DVL1-Wnt	Promotes hypoxia-induced pathological angiogenesis, exacerbating retinopathy and corneal neovascularization.	[80]
	1. HUVECs 2. C57BL/6J mice 3. CNV Model	↓	FTO↑	FTO-m6A demethylation-FAK (via YTHDF2)	Inhibits corneal neovascularization, alleviating pathological angiogenesis.	[83]
	1. Limbal stem cell-specific METTL3 knockout mice 2. Mouse Corneal Alkali Burn Model	——	METTL3↓	METTL3-m6A-AHNAK/DDIT4	Facilitates corneal injury repair, reducing neovascularization and inflammation.	[105]
	1. HUVECs 2. BALB/c mice 3. HSV-1 Induced Corneal Neovascularization Model	↑	METTL3↑	METTL3-m6A-LRP6-Wnt-VEGFA	Promotes corneal neovascularization.	[106]
Cataract	1. ARC Patient Lens Samples 2. HLE-B3 cells	↑	METTL3↑	METTL3/has_circ_0007905/miR-6749-3p/EIF4EBP1	Promotes apoptosis and proliferation of ARC cells.	[112]
	1. ALC Organization in DC Patients and ARC Patients 2. Human lens epithelial cells	↑	METTL3↑	METTL3-miR-4654-SOD2	Exacerbates oxidative stress and apoptosis in LEC, promoting lens opacification.	[113]
	1. Anterior lens capsule tissue in DC patients and normal patients without diabetes 2. Human lens epithelial cells	↑	METTL3↑	METTL3-ICAM-1	Promotes ICAM-1 expression, contributing to DC pathogenesis.	[114]
	1. Anterior lens capsule tissue in DC patients and normal patients without diabetes 2. Human lens epithelial cells	↑	RBM15↑	Ferroptosis	Promotes oxidative stress and ferroptosis in lens epithelial cells.	[115]
	RIR injury model mice	↓	METTL3↓	RIR- Autophagy Activation-m6A-METTL3-FoxO1 mRNA-FoxO1-Inhibition of Autophagy	Reduces RGC loss and retinal dysfunction caused by RIR injury.	[124]
	Ythdf2 cKO mice	——	YTHDF2↑	YTHDF2-Hspa12a/Islr2 mRNA-Hspa12a/Islr2-RGC	Decreases dendritic atrophy and neuronal loss.	[125]
	1. Primary HTFs 2. New Zealand White Rabbit	↑	METTL3↑	TGF-β1-smad3-METTL3-ECM	Increases HTFs proliferation and ECM accumulation, leading to scarring.	[128]
Uveitis	1. EAU Mouse Model 2. Human microglial cell line HMC3	↑	FTO↓	FTO-GPC4-TLR4-NF-xB	Enhances microglial activation and migration, exacerbating the inflammatory response.	[296]
	1. EAU Mouse Model 2. Human retinal pigment epithelial cell line ARPE-19	↑	FTO↓	FTO-ATF4- P-STAT3	Promotes secretion of inflammatory factors and degradation of tight junction proteins.	[133]
	1. EAU Mouse Model 2. BV2 microglial cell line	↓	YTHDC1↓	YTHDC1 -SIRT1 -STAT3 -M1	Upregulates pro-inflammatory phenotypic markers, worsening uveitis.	[134]
	Ocular tissue and T cell samples from the EAU model mice	↓	METTL3↓	METTL3-YTHDC2-ASH1L mRNA-ASH1L-IL-17/IL-23R-Th17	Suppresses Th17 cell responses, mitigating EAU.	[91]
	1. EAU Mouse Model 2. Mouse DCs	——	METTL3↑	METTL3-pri-miR-338-miR-338-3p-Dusp16-p38-DCs-Th17	Promotes Th17 cell generation and function, exacerbating EAU.	[136]
GO/TED	EMO specimens from 7 GO patients and 5 control subjects	↑	WTAP↑ ELF3↑ YTHDF2↑ YTHDC2↑	WTAP/YTHDF2/YTHDC2-IL-6/IL-18/TNF-α-NF-κB signaling pathway/Toll-like receptor signaling pathway/TNF signaling pathway	Triggers pro-inflammatory responses in EOMs, disrupting immune homeostasis and interfering with tissue remodeling and fibrosis of extraocular muscles.	[141]
	1. RAW 264.7 Mouse Macrophages Stimulated by LPS and Knockdown of YTHDF2	——	YTHDF2↓	YTHDF2-MAP2K4/MAP4K4 mRNA-NF-κB and MAPKsignaling pathway	Promotes expression of inflammatory factors, exacerbating macrophage inflammatory response.	[142]
	1. GD patients and healthy controls 2. PBMCs	↓	METTL3↓	METTL3-SOCS family	Affects RGC survival, optic nerve repair, and inflammatory response balance.	[145]
	GSE175399 (DNA methylation sequencing data), GSE186480 (tRFs expression data), and GSE185952 (mRNA, lncRNA, and circRNA expression data) in the GEO database	↑	——	M6A modification affects key pathways such as the IL-17 signaling pathway and cytokine receptor pathway	Promotes immune cell activation and inflammatory response.	[146]
TON	1. Establishment of the TON model in male Sprague-Dawley rats	↑	METTL3↑ WTAP↑ FTO↑ ALKBH5↑	METTL3/WTAP/FTO/ALKBH5-MAPK/NF-κB/TNF	Increases optic nerve injury, inflammation, and cellular homeostasis imbalance.	[210]
	Sciatic nerve compression and optic nerve compression models	↑	ALKBH5↓	ALKBH5-LPIN2	Promotes nerve regeneration, reducing optic nerve injury.	[214]
PM	1. SRAMP database 2. HSFs to construct myopic cell models	↓	METTL3↓	FOXM1-YTHDF2/METTL3-APOA1	Promotes HSF transdifferentiation into myofibroblasts, facilitating scleral remodeling.	[253]
	1. Form deprivation myopia models were established in pigmented guinea pigs and C57BL/6J mice 2. RF/6A cells	↓	METTL3↓	METTL3-Axin1/Arid1b mRNA-YTHDF2-Axin1/Arid1b	Facilitates choroidal vasculopathy.	[259]
	Anterior lens capsule in patients with simple nuclear cataract and nuclear cataract combined with high myopia	↑	METTL3↓ METTL14↑ FTO↓ ALKBH5↓ YTHDF1↓ YTHDF2↓	METTL3/METTL14/FTO/ALKBH5/YTHDF1/YTHDF2-ECM gene hypermethylation	Promotes abnormal ECM accumulation or change, affecting fundus anatomy and advancing high myopia pathology.	[10]
DR	1. STZ-induced DM mouse model 2. RMECs, rMCs	——	YTHDF2↓	KAT1-YTHDF2-ITGB1-FAK/PI3K/AKT	Abnormal vascular proliferation promotes inflammation and vascular leakage.	[157]
	1. STZ-induced DM mouse model 2. Pericytes	↑	METTL3↑	METTL3-YTHDF2-PKC-η/FAT4/PDGFRA	Inhibits pericyte survival, proliferation, and differentiation, compromising retinal vascular stability.	[163]
	1. Peripheral venous blood samples from T2D patients and healthy volunteers 2. Normal retinal cell line (ARPE-19)	——	METTL3↓	METTL3-miR-25-3p-/PTEN/Akt	Inhibits RPE cell proliferation, accelerating apoptosis and pyroptosis.	[166]
	Normal retinal cell line (ARPE-19)	↑	FTO↑	miR-192-FTO-NLRP3	Increases pyroptosis of RPE cells.	[168]
	1. STZ-induced DM mouse model 2. HRMECs	↓	METTL3↓	METTL3-SNHG7-KHSRP-MKL1-EndoMT	Increases pyroptosis of REP cells.	[171]
	1. Patients with PDR due to T1D or T2D 2. STZ-induced murine model of T1D and high-fat diet combined with STZ-induced T2D 3. EC FtoΔ/Δ mouse model 4. HRMECs	↓	FTO↑	FTO-TNIP1-NF-κB Pathway-IL-1β and IL-18 Release	Retinal vascular leakage and acellular capillary formation.	[88]
	1. STZ-induced DM mouse model 2. OIR mouse model 3. HUVECs	↓	FTO↑	FTO-CDK2	Aggravates DM-induced angiogenesis, microvascular leakage, inflammation, and neurodegeneration.	[90]
	1. THP-1, HRMECs 2. STZ-induced DM mouse model	↑	FTO↓	FTO-FGF2-PI3K/AKT	Polarizes macrophages toward M1, worsening the inflammatory response.	[86]
	1. STZ-induced DM rat model 2. Microglial cell line BV2	——	ALKBH5↓	ALKBH5-A20 (TNFAIP3)	Affects microglial polarization state, leading to aggravated inflammation.	[178]
ROP	1. OIR mouse model 2. GO and KEGG analysis 3. ceRNA Network Analysis	——	——	1. Association between m6A modification levels and circRNA expression levels in OIR retinas. 2. circRNA-miRNA-mRNA network was constructed	Provides new insights into the molecular mechanisms of m6A-modified retinal neovascularization.	[183]
	OIR mouse model	——	——	M6A modification changes in mRNA and lncRNA in OIR retina	Investigates the potential role of m6A modification in retinal neovascularization.	[184]
RP	Normal retinal cell line (ARPE-19)	↓	METTL1↓	METTL14-MAP2-NEUROD1	Decreases RPE phagocytic ability, disrupts tight junctions, increases apoptosis, and induces cell cycle arrest.	[189]
PVR	HTERT RPE-1 cell line	——	METTL3↓ YTHDF1↑	TGF-β2-METTL3/YTHDF1 ↑ -EMT	Induces EMT in RPE cells, promoting PVR fibrosis.	[196]
	1. Tissue samples from PVR patients and normal donor eyes 2. Normal retinal cell lines (ARPE-19) 3. rats with intravitreal METTL3 overexpression	↓	METTL3↓	METTL3-Wnt/β-catenin signaling pathway	Promotes RPE cell EMT, enhancing proliferation and migration, accelerating proliferative membrane formation.	[198]
	HTERT RPE-1 cell line	——	——	MeCP2 treatment: 9,041 m6A peaks downregulated, 4 upregulated	Facilitates EMT process in RPE cells.	[197]
AMD	1. Primary mouse RPE cells and the human RPE cell line ARPE-19 2. C57BL/6 mice injected with Aβ1-40 and the FTO inhibitor MA1 in the vitreous	——	FTO↑	2.FTO-PKA/CREB Signaling Pathway	Exacerbates RPE cell degeneration.	[205]
CM	1. 88 ocular melanoma tissues and 28 normal melanocyte tissues 2. Multiple ocular melanoma cell lines and normal melanocyte cell lines 3. Nude mice that were subcutaneously injected with melanoma cells	↓	YTHDF1↑ METTL3↓	METTL3-YTHDF1-HINT2	Accelerates melanoma progression, leading to poor prognosis.	[220]
	1. 9 CM patients in the GEO database 2. 41 CM samples and 11 normal melanocytic nevus samples clinically	↓	FTO↑	FTO-EGR1/VEGFA	Promotes tumor angiogenesis mediated by CAFs.	[222]
UM	1. 88 ocular melanoma tissues and 28 normal melanocyte tissues 2. multiple ocular melanoma cell lines and normal melanocyte cell lines 3. Nude mice that were subcutaneously injected with melanoma cells	↓	YTHDF1↑ METTL3↓	METTL3-YTHDF1-HINT2	Accelerates melanoma progression, leading to poor prognosis.	
	3 UM tissues and 3 normal choroid tissues	↓	FTO↑	FTO-ATG5	Inhibits autophagy in UM cells.	[231]
	1. CM tissue and normal choroid tissue samples from 36 patients 2. Two human CM cell lines, OCM1 and MUM-2B 3. Nude mice injected with CM cells into the caudal vertebrae	——	METTL4↑	METTL4-RUNX2 mRNA-Wnt/β-catenin signaling pathway	Facilitates CM cell migration and invasion.	[233]
	1. GEPIA database analysis 2. MuM-2B and C918 3. Nude mice that were subcutaneously injected with ALKBH5 stably knocked down C918 cells	——	ALKBH5↑	ALKBH5-FOXM1 mRNA	Promotes UM cell proliferation and inhibits apoptosis.	[234]
	1. UM cell line 2. 11 primary UM samples	↑	METTL3↑	METTL3-c-Met/Akt signaling pathway	Promotes UM cell proliferation, colony formation, migration, and invasion.	[235]
	1. 5 UM tissues and 5 normal uveal melanocyte tissue samples 2. 6 Ocular melanoma cell lines and 1 retinal pigment epithelial cell line	——	IGF2BP3↑	circ_0053943-IGF2BP3/EGFR	Promotes UM cell proliferation, migration, and invasion.	[236]
	Pan-Cancer Datasets for TCGA, TARGET, and GTEx	——	ZC3H13↑	ZC3H13-ACKR2	Significantly associated with longer OS and PFI in UM patients.	[237]
	The TCGA database obtains RNA-seq and clinical data from UM patients	——	RBM15B↑	1.LINC00665/hsa-let-7b-5p/RBM15B axis 2.LINC00638/hsa-miR-103a-3p/RBM15B axis	UM patients had significantly longer OS, DSS, and PFI.	[238]
	The TCGA database obtains RNA sequencing transcriptome data and clinical data from 80 UM patients	——	RBM15B↑ YTHDF3↑ IGF2BP2↑	LncRNA	Highly expressed RBM15B and IGF2BP2 are associated with better prognosis, whereas highly expressed YTHDF3 is associated with worse prognosis.	[239]
RB	1. GEO database and miRDB database 2. with RB cell lines (WERI-Rb-1 and Y 79) and normal retinal cell lines (ARPE-19)	——	METTL14↑	METTL14-LINC00340-Notch signaling pathway	Promotes RB cell growth and inhibits apoptosis.	[244]
	1. GSE24673 dataset obtained from the GEO database 2. Multiple RB cell lines	——	IGF2BP3↑	IGF2BP3-USP49-SIRT1	Aggravats RB resistance to carboplatin CBP.	[297]
	1. GSE208143 dataset 2. RB cell line 3. Nude mouse models injected intraocularly with sh-FTO and E2F3	↓	FTO↑	FTO-YTHDF2-E2F3	Promotes the malignant progression of RB cells.	[246]
	1. 30 RB patients and normal tissue samples 2. RB cell lines (WERI-Rb-1 and Y 79)	——	METTL14↑	METTL14-CDKN2A-p53 signaling pathway	Promotes RB cell proliferation and inhibit apoptosis.	[298]
	1. mRNA sequencing data 2. RB cell lines (WERI-Rb-1 and Y 79) and normal retinal cell lines (ARPE-19)	——	YTHDF1↑	MYCN-YTHDF1-CDK5R1	Promotes the proliferation and tumor growth of RB cells	[248]
	1. RB tissue samples and two RB cell lines (Y 79 and WERI-Rb-1) 2. Subcutaneous injection of RB cells with METTL3 knockdown in nude mice	——	METTL3↑	METTL3-PI3K/AKT/mTOR	Promotes migration and invasion of RB cells	[191]

*ACKR2* atypical chemokine receptor 2, *AHNAK* AHNAK nucleoprotein, *Akt* protein kinase B, *ALC* anterior lens capsule, *ALKBH5* AlkB homolog 5, *ARC* age-related cataract, *ARPE-19* adult retinal pigment epithelial cell line, *BACE2* beta-secretase 2, *C918* C918 cell line, *CAFs* cancer-associated fibroblasts, *CBP* carboplatin, *CDK2* cyclin-dependent kinase 2, *CDKN2A* cyclin-dependent kinase inhibitor 2A, *CM* conjuncval melanoma, *CNV* choroidal neovascularization, *DC* diabetic cataract, *DCs* dendritic cells, *DDIT4* DNA-damage-inducible transcript 4, *DM* diabetes mellitus, *DR* diabetic retinopathy, *DRG* dorsal root ganglion, *DVL1* disheveled segment polarity protein 1, *E2F3* E2F transcription factor 3, *EAU* experimental autoimmune uveitis, *EC* FtoΔ/Δ endothelial cell-specific Fto-deficient mice, *EGR1* early growth response 1, *EMT* epithelial-mesenchymal transition, *EndoMT* endothelial-mesenchymal transition, *EOMs* extraocular muscles, *FAK* focal adhesion kinase, *FAT4* FAT atypical cadherin 4, *FOXM* forkhead box M1, *FTO* fat mass and obesity-associated protein, *FTO* FTO α-ketoglutarate-dependent dioxygenase, *GEPIA* gene expression profiling interactive analysis, *GTEx* the genotype-tissue expression, *HINT2* histidine triad nucleotide-binding protein 2, *HRMECs* human retinal microvascular endothelial cells, *HSFs* human scleral fibroblasts, *HTFs* human tenon’s capsule fibroblasts, *HUVECs* human umbilical vein endothelial cells, *IGF2BP3* insulin-like growth factor 2 mRNA-binding protein 3, *IL-1β* interleukin-1 beta, *ITGB1* integrin beta 1, *KAT1* lysine acetyltransferase 1, *LEC* lens epithelium cell, *LncRNA* long non-coding RNA, *LRP6* low-density lipoprotein receptor-related protein 6, *M6A* N6-methyladenosine, *MAP2* microtubule-associated protein 2, *MCP-1* monocyte chemoattractant protein 1, *MeCP2* methyl-CpG binding protein 2, *METTL3* methyltransferase-like 3, *MKL1* megakaryocytic leukemia 1, *MuM-2B* malignant uveal melanoma cell line 2B, *MYCN* N-Myc proto-oncogene protein, *NEUROD1* neuronal differentiation 1, *NF-κB* nuclear factor kappa-B, *NLRP3* nucleotide-binding domain leucine-rich repeat family protein 3, *Notch* Notch signaling pathway, *OIR* oxygen-induced retinopathy, *ONC* optic nerve compression, *OS* overall survival, *PBMCs* peripheral blood mononuclear cells, *PDGFRA* platelet-derived growth factor receptor alpha, *PFI* progression-free interval, *PI3K* phosphatidylinositol 3-kinase, *p-IκB/IκB* phosphorylated/inhibitor of nuclear factor kappa-B, *PKC-η* protein kinase C eta, *p-p65/p65* phosphorylated/nuclear factor kappa-B subunit p65, *PRV* proliferative vitreoretinopathy, *PTEN* phosphatase and tensin homolog, *PVR* proliferative vitreoretinopathy, *PXG* pseudoexfoliation glaucoma, *RB* retinoblastoma, *RIR* retinal ischemia-reperfusion, *rMCs* retinal Müller cells, *RMECs* retinal microvascular endothelial cells, *ROP* retinopathy of prematurity, *RP* retinitis pigmentosa, *RPE* retinal pigment epithelium, *SIRT1* sirtuin 1, *SNC* sciatic nerve compression, *SNHG7* small nucleolar RNA host gene 7, *STZ* streptozotocin, *T1D* type 1 diabetes mellitus, *T2DM* type 2 diabetes mellitus, *TARGET* therapeutically applicable research to generate effective treatments, *TCGA* The Cancer Genome Atlas, *TGF-β2* transforming growth factor beta 2, *THP-1* human acute monocytic leukemia cell line, *TMEM38B* transmembrane protein 38B, *TNFAIP3* tumor necrosis factor-α induced protein 3, *TNIP1* TNFAIP3 interacting protein 1, *TRAF6* TNF receptor-associated factor 6, *UM* uveal melanoma, *USP49* ubiquitin-specific peptidase 49, *VEGFA* vascular endothelial growth factor A, *YTHDF2* YTH domain family 2, *ZC3H13* zinc finger CCCH-type containing 13.

Despite the compelling evidence linking m6A modifications to the pathogenesis of various ocular diseases, it is crucial to interpret these findings, particularly their therapeutic implications, with caution. The current body of evidence is predominantly derived from in vitro and in vivo preclinical models. While these studies provide invaluable mechanistic insights and identify promising therapeutic targets, they cannot fully recapitulate the complexity of human disease. Significant challenges, including the development of targeted and safe delivery systems for ocular tissues, potential off-target effects, and long-term efficacy and safety profiles, must be thoroughly addressed before clinical translation can be contemplated. Therefore, while the modulation of m6A represents a frontier of great scientific interest, its near-term clinical relevance for treating eye diseases should not be overstated. Future research must prioritize the transition from foundational discovery to rigorous translational studies, which are essential for bridging the gap between promising preclinical data and tangible clinical applications.
